# The antimicrobial peptide LI14 combats multidrug-resistant bacterial infections

**DOI:** 10.1038/s42003-022-03899-4

**Published:** 2022-09-07

**Authors:** Jingru Shi, Chen Chen, Dejuan Wang, Zhiqiang Wang, Yuan Liu

**Affiliations:** 1 College of Veterinary Medicine, Yangzhou University, Yangzhou, 225009 China; 2 Jiangsu Co-innovation Center for Prevention and Control of Important Animal Infectious Diseases and Zoonoses, Yangzhou University, Yangzhou, 225009 China; 3 Joint International Research Laboratory of Agriculture and Agri-Product Safety, the Ministry of Education of China, Yangzhou University, Yangzhou, 225009 China; 4 Institute of Comparative Medicine, Yangzhou University, Yangzhou, 225009 China

**Keywords:** Microbiology, Drug discovery

## Abstract

The prevalence of multidrug-resistant (MDR) pathogens raises public fears of untreatable infections and represents a huge health risk. There is an urgent need to exploit novel antimicrobial agents. Due to the unique mechanisms, antimicrobial peptides (AMPs) with a low probability to achieve resistance are regarded as potential antibiotic alternatives to address this issue. Herein, we develop a panel of synthetic peptide compounds with novel structures based on the database filters technology (DFT), and the lead peptide LI14 shows potent antibacterial activity against all tested drug-resistant bacteria. LI14 exhibits rapid bactericidal activity and excellent anti-biofilm and -persisters activity, simultaneously showing a low propensity to induce resistance. Moreover, LI14 shows tolerance against pH, temperatures, and pepsin treatment, and no detectable toxicity both in vitro and in vivo. Mechanistic studies revealed that LI14 induces membrane damage by targeting bacterial-specific membrane components and dissipates the proton motive force (PMF), thereby resulting in metabolic perturbations and the accumulation of toxic metabolic products. Furthermore, LI14 sensitizes clinically relevant antibiotics against MDR bacteria. In animal models of infection, LI14 or combined with antibiotics are effective against drug-resistant pathogens. These findings suggest that LI14 is a promising antibiotic candidate to tackle MDR bacterial infections.

## Abstract (web summary)

A synthetic peptide LI14 demonstrates potent antibacterial activity against drug-resistant bacteria in vitro and in vivo by inducing membrane damage and disrupting membrane potential leading to metabolic perturbation.

## Introduction

The increasing crisis of antimicrobial resistance in pathogenic bacteria exacerbates untreatable systemic infections in the clinic, leading to high mortality in patients and economic loss^1^. Drug-resistant ESKAPE (*Enterococcus faecium*, *Staphylococcus aureus*, *Klebsiella pneumoniae*, *Acinetobacter baumannii*, *Pseudomonas aeruginosa*, and *Enterobacter* species) bacteria are the main causes of the majority of nosocomial infection worldwide^2,3^. Alarmingly, novel plasmid-encoded mobile antibiotic resistance genes (ARGs) such as *bla*_NDM-1_, *mcr-1*, and *tet*(X3/X4) were found to mediate bacterial resistance to carbapenems^4^, colistin^5^, and tigecycline^6,7^, respectively, which are considered the last resort for the treatment of Gram-negative bacteria relevant infections. In addition to the acquired resistance by horizontal gene transfer and chromosomal mutation, the formation of biofilm in these bacteria^8^, a collection of microbial communities encased in an extracellular matrix, further aggravates this crisis with a substantially reduced antibiotic susceptibility^9^ and leaves few choices for clinicians. Meanwhile, changes in the bacterial metabolic state also have an important impact on antibiotic potency^10^. For instance, bacterial persisters are a small stochastically formed subpopulation of low-energy bacterial cells and are highly tolerant to traditional antibiotics^11^, which are responsible for chronic and recurrent infections^12^. Facing these challenges, there is a dire need to identify novel and promising compounds to combat these drug-resistant or tolerant pathogens.

Antimicrobial peptides (AMPs), also recognized as host defense peptides, are bioactive small proteins produced by multicellular organisms as immune defenses to fight other harmful microorganisms^13,14^. As one of the most promising antibacterial agents, contrary to conventional antibiotics, the majority of AMPs act by disrupting bacterial membrane, thus usually along with a lower resistance level^15,16^. On account of the potent antibacterial activity, unique mechanism and rare resistant mutants, AMPs are gaining traction as potential treatment options against drug-resistant bacterial infections^17,18^. Usually, natural AMPs are not always well-optimized for direct antimicrobial activity, and it is possible that a variety of moderately active peptides with concomitant immunomodulatory properties act effectively when they are delivered directly to infection sites^19^. By contrast, some synthetic AMPs derived from computer-aided de novo creation or chemical improvement of existing natural AMPs display extremely potent activity in fighting against MDR pathogens^20^. For example, SAAP-148 optimized from human AMPs LL-37 showed excellent plasma stability and bactericidal activity against MDR pathogens, simultaneously could effectively combat biofilm-producing bacteria and persister cells^21^. Furthermore, MSI-1 was obtained by truncating MSI-78, which exhibited improved activity and safety compared to its parent AMPs^22^. Despite these ongoing efforts, few synthetic AMPs were approved for clinical use, partly due to the weak stability under physiological conditions or non-specific toxicity in vivo.

In this study, we performed a rational drug design using database filters technology (DFT) and obtained a novel synthetic antibacterial peptide termed LI14. Furthermore, we evaluated its systematic effectiveness against MDR pathogen in vitro and in animal models of infection, as well as its stability, safety, mechanisms of action, and synergistic activity with clinically relevant antibiotics. Our work highlights the therapeutic potential of LI14 peptide in the fight against infections caused by antibiotic-resistant bacteria.

## Results

### Database-guided peptide design and characterizations of designed peptides

**Fig. 1:**
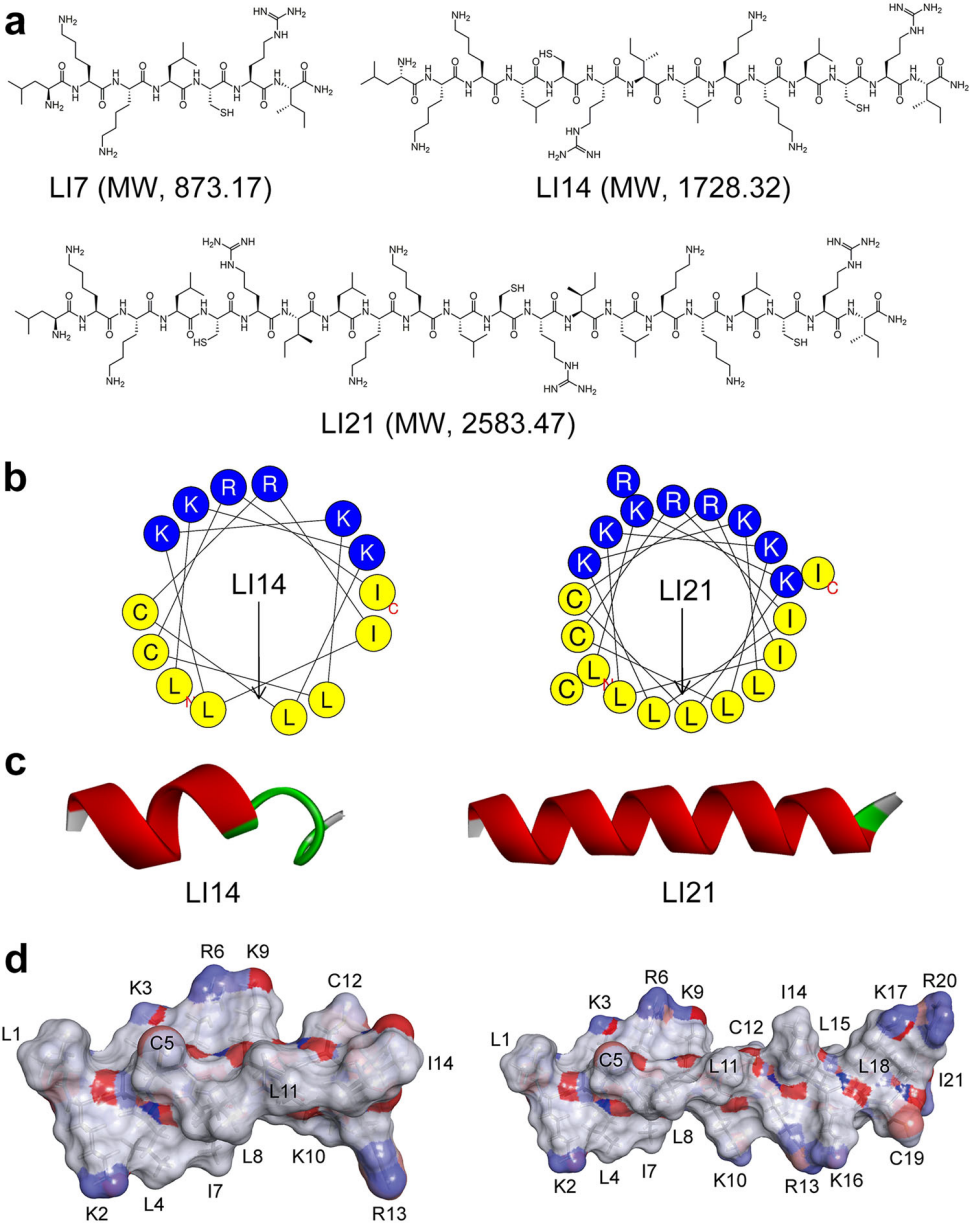
Design and characterization of novel antimicrobial peptides. **a** Chemical structures of three LI peptides. **b** Helical wheel projections of peptides using HeliQuest analysis (http://heliquest.ipmc.cnrs.fr/cgi-bin/ComputParamsV2.py). Amino acids in blue are positively charged, while in yellow are hydrophobic. The length of the arrow represents the relative hydrophobic moments in the figure, which are positively correlated. **c** Three-dimensional structure projections of peptides. I-TASSER (http://zhanglab.ccmb.med.umich.edu/I-TASSER/) was used to forecast the three-dimensional structure projection of LI14 and LI21 peptides. The different colors represent various secondary structure types: red, helix; green, turn; gray, coil. **d** Potential surfaces of LI14 (left) and LI21 (right) peptides.

Antibacterial peptide database (APD) includes all kinds of peptides antibiotics information, which laid the foundation for peptide screening and the development of database-filtering technology (DFT)^23,24^. DFT is able to utilize the most likely parameters derived from a set of preclinical- and clinical-phase AMPs to design novel and active peptides^25^. Here, we aimed to design a set of broad-spectrum AMPs. To identify broad-spectrum AMPs with great systemic efficacy in murine models of infection, we applied DFT to assist the design of promising AMPs. To simplify the design, seven high-frequency amino acids from broad-spectrum peptides: Glycine (G), Lysine (K), Leucine (L), Alanine (A), Isoleucine (I), Cysteine (C), and Arginine (R) were screened^19^. Considering that the side chain of G and A has a minimal effect on the α-helical structure of peptides, thus these two amino acids were discarded in the following design. To generate the hydrophobic core in the amphipathic α-helices, L residues are placed at the 1 and 4 positions. Compared with the LK, LR, LKR, and LRR, the combination LKK showed high frequency and priority. On the basis of the above steps, a novel heptad repeat sequence template (LKKLCRI)n (*n* = 1, 2, or 3) was developed and three corresponding AMPs termed LI7 (LKKLCRI-NH_2_), LI14 ((LKKLCRI)_2_-NH_2_), and LI21 ((LKKLCRI)_3_-NH_2_) were obtained (Fig. 1a). To improve the stability of peptides, all synthesized peptides were modified with C-termini amidation (Supplementary Table 2).

Helical wheel projections results showed that amino acids with high hydrophobicity and those with low hydrophobicity exhibited obvious bipolarity distribution, implying that these peptides may adopt a typical helical structure (Fig. 1b). Consistently, the predicted 3D structures and the surface membrane potential distribution showed that LI14 and LI21 peptides had perfect hydrophobic and cationic faces (Fig. 1c, d). Moreover, the results from physiological properties calculation (Supplementary Table 2) showed that LI14 and its derivatives showed typical cationic and high hydrophobicity, and all the purity of them were greater than 95%, manifesting that they were all obtained precisely.

As shown in Table 1, LI14 and LI21 displayed superior antibacterial activity against both Gram-positive and Gram-negative bacteria, whereas LI7 showed weak bacteriostatic activity against all test strains. Considering that L17 is only made up of seven amino acids, we speculate that it was too short to form the specific secondary structures, which may hinder its activity. Interestingly, LI14 showed broad-spectrum and strong antibacterial activity against all MDR pathogens with MIC values ranging from 1 to 64 μg/mL, involving methicillin-resistant *Staphylococcus aureus* (MRSA), vancomycin-resistant Enterococci (VRE), carbapenem-resistant Enterobacteriaceae (CRE), MCR-positive *E. coli* (MCRPEC) and tigecycline-resistant pathogen, which was comparable to that of some antibiotics used clinically. To better assess the impact of clinically important resistance determinants on the activity of LI14, we determined the antibacterial activity of LI14 against a series of engineered bacteria, which carries *bla*_NDM_, *mcr*, or *tet*(X4) and confers resistance to carbapenems, colistin or tigecycline, respectively. Excitingly, LI14 displayed equivalent activity for *E. coli* DH5α and engineered resistant bacteria, indicating that the known resistance genes had no effect on the action of LI14.

**Table 1:** Antimicrobial spectrum of engineered LI peptides (MIC, μg/mL).

Organisms and genotypes	LI7	LI14	LI21	Meropenem	Colistin	Tigecycline
*Gram-positive bacteria*						
*S. aureus* ATCC 29213	>128	8	32	<0.0625	16	0.125
MRSA T144	>128	4	32	1	16	0.25
MRSA 1518	>128	64	32	0.25	16	0.25
MRSA 1530	>128	64	32	0.25	16	1
*S. aureus* 215 (*cfr* + LZD^R^)	64	1	16	1	16	0.25
*S. aureus* G16 (RIF^R^)	32	1	16	2	1	<0.0625
*E. faecalis* A4 (VRE, VanA)	>128	8	32	>64	64	<0.0625
*E. faecalis* 1F-1	>128	64	64	8	>64	16
*E. faecium* 5F-10	>128	64	64	8	>64	<0.0625
*Gram-negative bacteria*						
*E. coli* ATCC 25922	>128	8	64	0.03	0.5	0.125
*E. coli* B2 (*bla*_NDM-5_ + *mcr-1*)	>128	4	64	32	8	2
*E. coli* C3 (*bla*_NDM-1_)	>128	4	32	8	<0.125	2
*E. coli* G6 (*bla*_NDM-5_)	>128	4	16	64	0.5	2
*E. coli* G92 (*mcr-1*)	>128	8	32	<0.125	4	4
*E. coli* CP131 (*mcr-3*)	>128	4	16	<0.125	2	2
*E. coli* B3-1 (*tet*(X4))	>128	8	32	<0.0625	0.0625	32
*E. coli* 1F28 (*tet*(X4))	>128	8	32	0.25	0.125	16
*S. enteritidis* ATCC 13076	>128	8	32	<0.0625	0.25	0.125
*A. baumannii* ATCC 19609	>128	8	32	<0.0625	0.125	0.25
*A. baumannii* C222 (*tet*(X6))	>128	16	32	<0.125	<0.125	64
*P. aeruginosa* PA14	>128	16	32	32	1	<0.0625
*K. pneumoniae* ATCC 700603	>128	32	64	<0.0625	1	0.5
*K. pneumoniae* D120 (*mcr-8*)	>128	32	32	<0.0625	4	0.25
*P. cibarius* HNCF44W (*bla*_NDM-1_ + *tet*(X6))	>128	8	32	>16	>256	64
*Engineered strains*						
*E. coli* DH5α (PUC19)	>128	2	16	<0.125	<0.125	1
*E. coli* DH5α (PUC19-*tet*(X4))	>128	2	16	<0.125	<0.125	64
*E. coli* DH5α (PUC19-*bla*_NDM-1_)	>128	2	16	32	<0.125	1
*E. coli* DH5α (PUC19-*bla*_NDM-5_)	>128	4	16	64	<0.125	1
*E. coli* DH5α (PUC19-*bla*_NDM-9_)	>128	2	16	64	<0.125	1
*E. coli* DH5α (PUC19-*mcr-1*)	>128	2	16	<0.125	2	1

*MRSA* methicillin-resistant *Staphylococcus aureus*, *RIF*^*R*^ rifampicin-resistant, *VRE* vancomycin-resistant Enterococci.

To elucidate the structure-activity relationship and active center of LI14 peptide, we conducted an alanine scanning library and obtained 14 Ala substitutes (Supplementary Table 2). Interestingly, we found that LI14-A_6_ almost lost activity against four tested bacteria (Supplementary Table 3), suggesting the Arg at position 6 was necessary for the antibacterial activity of LI14 peptide. The following helical wheel projection (Supplementary Fig. 1) and CD spectrum in different solutions (Supplementary Fig. 2) results indicated that Ala substitution of Arg at position 6 of LI14 strongly disrupted its secondary structure. Besides, our results indicated that the cyclic peptides LI14 (S-S) showed comparable antibacterial activity with linear LI14, suggesting that the cyclization of LI14 (S-S) via disulfide bond did not affect its antibacterial activity. To reduce the production costs, linear LI14 was selected as the lead peptide for further studies.

### Rapid bactericidal and anti-biofilm activity of LI14 peptide

**Fig. 2:**
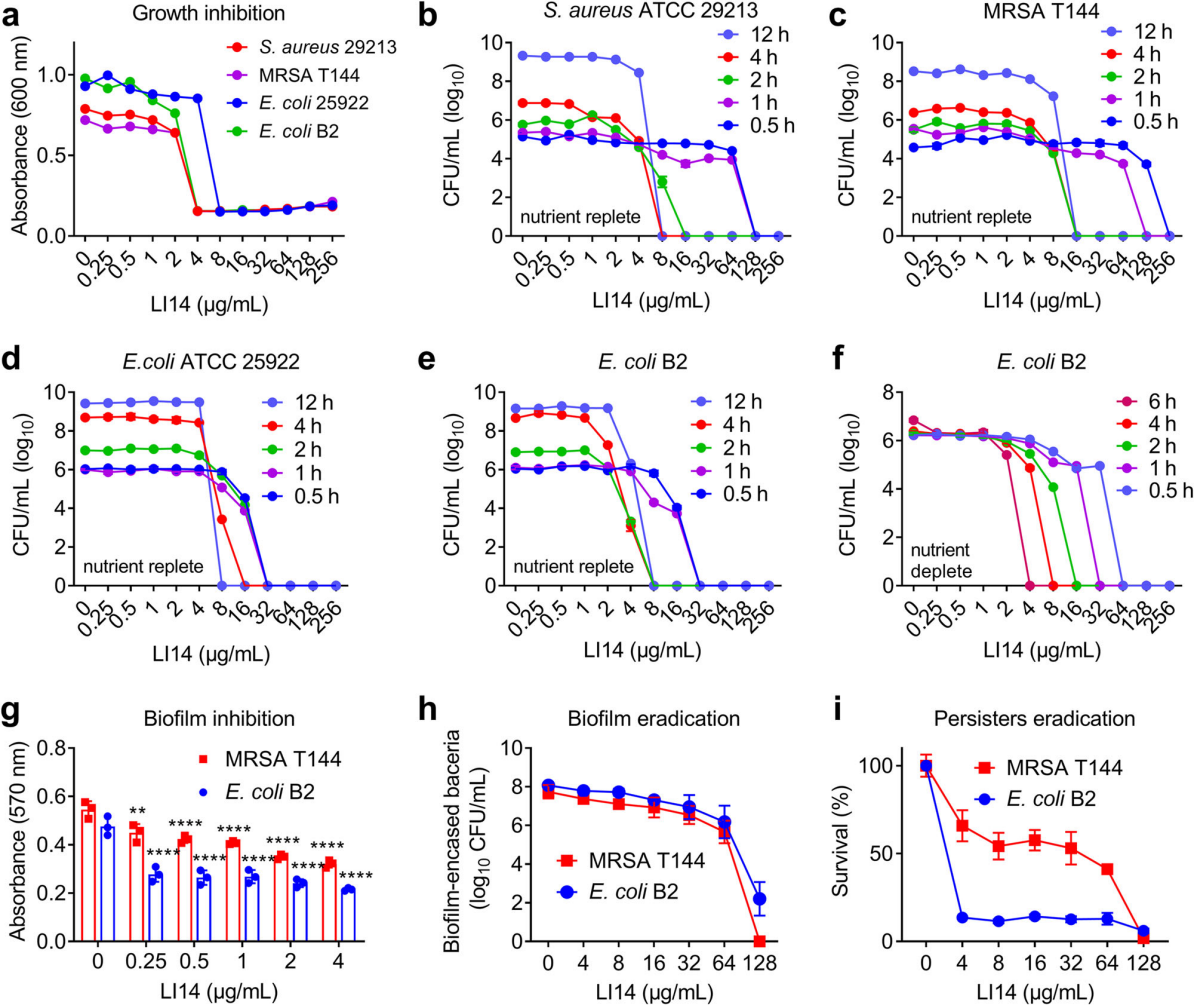
LI14 peptide is a potent broad-spectrum bactericidal agent. **a** Growth inhibition of four strains involves *S. aureus* ATCC 29213, MRSA T144, *E. coli* ATCC 25922, and *E. coli* B2 after incubation with various concentrations of LI14 peptide for 18 h at 37 °C. **b**–**e** Killing of *S. aureus* ATCC 29213 (**b**), MRSA T144 (**c**), *E. coli* ATCC 25922 (**d**), and *E. coli* B2 (**e**) in MHB broth in the presence of difference concentrations of LI14 peptide after incubation for 0.5 to 12 h at 37 °C. The initial cell density of ~10^5^ CFUs/mL for Gram-positive bacteria, ~10^6^ CFUs/mL for Gram-negative bacteria. **f** Killing of *E. coli* B2 in PBS (nutrient depletion) treated by the varying concentrations of LI14 after incubation for 0.5–6 h at 37 °C. **g** Biofilm inhibition ability of sub-inhibitory concentrations of LI14 peptide against two tested strains, MRSA T144 and *E. coli* B2. One-way ANONA was used to determine the statistical significance (**P < 0.01 and ****P < 0.0001). **h**, **i** LI14 efficiently eradicates established biofilms (**h**) and persister cells (**i**) of MRSA T144 and *E. coli* B2. Experiments were conducted with three biological replicates and data were shown as mean ± SD.

In light of the growth inhibition properties of LI14 peptide against both standard and clinical strains (Fig. 2a), we further performed the time-killing curves to evaluate whether LI14 peptide is a bactericidal compound^26^. In a nutrient-rich MHB broth with an initial cell density of ~10^5^ CFUs/mL for Gram-positive bacteria, and ~10^6^ CFUs/mL for Gram-negative bacteria, we found that LI14 induced obvious bacterial cell death with a time- and concentration-dependent manner (Fig. 2b–e). Specifically, 32 μg/mL LI14 could elucidate all *E. coli* strains during 0.5 h, and 8 μg/mL LI14 could completely eliminate the three tested strains at 12 h, except for MRSA T144. In the following study, we evaluated whether LI14 has bactericidal activity against bacteria under growth-metabolic inhibition, which displayed high tolerance to antibiotic treatment^27^. Interestingly, we observed that LI14 conserved bactericidal activity in nutrient-depleted buffer (PBS), and it is almost as effective as in nutritional conditions (Fig. 2f). This is in distinct contrast to the conventional bactericidal antibiotic ampicillin, which possesses strong bactericidal activity in nutritional conditions but is incapable of eradicating bacteria in metabolically suppressed states^26^. Resistance development to new antibacterial agents has been a major concern, thus we assessed the ability of Gram-positive and Gram-negative pathogens to develop resistance to LI14 peptide. As shown in Supplementary Fig. 3, after 80 serial passages in the presence of sub-inhibitory concentrations of LI14, the relative MIC values of LI14 against *S. aureus* ATCC 29213 and *E. coli* ATCC 25922 were only enhanced by 8-fold, while exposure to the rifampicin or ciprofloxacin resulted in a rapid increase in MIC by 1024- and 256-fold, respectively, indicating that LI14 is less prone to developing resistance compared to conventional antibiotics.

Bacterial biofilms play a vital role in the development of their pathogenicity and drug resistance, and are usually associated with chronic infections^28^. We next evaluated the anti-biofilm activity of LI14 peptide using crystal violet staining. As shown in Fig. 2g and h, LI14 dose-dependently restrained the generation of biofilms at the sub-inhibitory concentrations and almost eradicated all established biofilms at 128 μg/mL. We further evaluated the bactericidal activity of LI14 to persisters, which can withstand the killing of high-concentrations of antibiotics. Results indicated that LI14 concentration-dependently eliminated persisters, especially for *E. coli* B2 persisters, and the removal rate of the persisters reached more than 99% at 128 μg/mL of LI14 (Fig. 2i). Altogether, these data suggest that LI14 is a potent bactericidal peptide antibiotic for both drug-resistant pathogens, biofilms and persisters.

### Desirable stability of LI14 peptide

The stability of AMPs is one of the decisive factors accounting for its in vivo effectiveness^29^, thus we tested the stability of LI14 peptide under the circumstances of salt ions, serum, DMEM medium, and proteases. As shown in Supplementary Table 4, the addition of monovalent cations (Na^+^ and K^+^) did not influence the activity of LI14, while the addition of divalent cation Mg^2+^, 10% DMEM and serum slightly impaired the activity of LI14 with MIC values increased by 4–8-fold. Divalent ions such as Mg^2+^ can form a salt ion bridge of lipid A through electrostatic interaction, which plays a role in outer membrane stabilization. Thus, we reasoned that the molecular mechanism of LI14 might be linked to bacterial membrane disruption^30^. Furthermore, thermal, pH and protease stability studies showed that LI14 was stable in the 40–100 °C and pH from 2 to 12, while at 121 °C, its antibacterial activity decreased by 4-fold (Supplementary Table 4). Interestingly, LI14 conserved full activity after incubation with pepsin, while trypsin and papain abolished the activity of LI14 peptide (Supplementary Table 4), indicating LI14 had a strong resistance to the proteolytic effect of pepsin. This may be relevant to amino acid compositions of LI14, as pepsin usually hydrolyzed aromatic amino acids, trypsin and papain usually hydrolyzed arginine, lysine, etc. Overall, these data suggest that LI14 peptide shows great thermal, pH and pepsin stability.

### LI14 peptide has no detectable toxicity both in vitro and in vivo

Hemolysis and cytotoxicity to mammalian cells are severe barriers to prevent the translational application of peptides^31^, we sought to evaluate the safety of LI14 peptide both in vitro and in vivo. We first evaluated the in vitro toxicity of LI14 and its derivatives to mammalian red blood cells (RBCs). We found that LI14 had a dispensable and the lowest hemolytic activity (lower than 5% at 128 µg/mL), while LI21, LI14-A3, and LI14-A13 showed moderate hemolytic activity with 10–40% at 128 µg/mL (Supplementary Fig. 4). These results implied that LI14 had lower toxicity to mammalian cells. Then, we assessed the in vivo toxicity of LI14 peptide in mice through daily intraperitoneal injection of LI14 peptide for 6 days at a dose of 10 mg/kg (Supplementary Fig. 5a). During one week of intraperitoneal injection, we did not observe murine behavior changes or body weight loss (Supplementary Fig. 5b). Whole-blood cell profiles and serum biochemical index detection showed no significant difference compared with the control group (Supplementary Fig. 5c, d). Moreover, after one week of intraperitoneal injection, the mouse organs including the heart, liver, spleen, lung, and kidney were taken for H.E. staining, and the results showed that there was no significant difference in histological morphology (Supplementary Fig. 5e). These data indicate that LI14 peptide is a safe and potent antibacterial compound.

### LI14 peptide targets multiple components of bacterial membrane

**Fig. 3:**
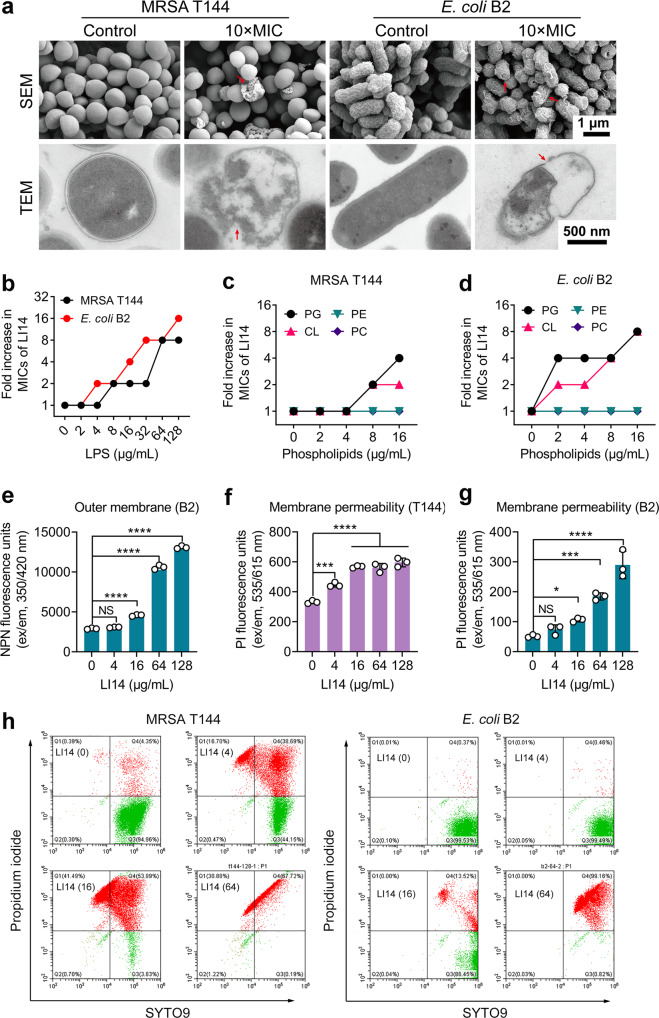
LI14 peptide disrupts membrane integrity by targeting membrane components. **a** SEM and TEM micrographs of MRSA T144 and *E. coli* B2 after exposure to LI14 peptide (10-fold MIC) for 1 h (Scar bars: Upper, 1 μm; Lower, 500 nm). **b**–**d** Exogenous LPS (**b**) and two phospholipids (PG and CL, **c** and **d**) abolished the antibacterial activity of LI14 peptide. Checkerboard microdilution experiments were applied to calculate the MIC changes of LI14 against MRSA T144 and *E. coli* B2 in the presence of various doses of LPS (0–128 μg/mL), PC, PE, PG, and CL (0–16 μg/mL). **e** LI14 peptide enhances the outer membrane permeability of *E. coli* B2. The outer membrane permeability was determined using 1-*N*-phenylnaphthylamine (NPN) with the excitation/emission wavelength at 350 nm/420 nm. **f**, **g** Increased inner membrane permeability of MRSA T144 (**f**) and *E. coli* B2 (**g**) after treatment of LI14 peptide. Propidium iodide (PI) was used to measure membrane permeability, with an excitation/emission wavelength of 535 nm/615 nm. **h** LIVE/DEAD *Bac*Light viability assay of MRSA T144 and *E. coli* B2 after treatment of different does of LI14 peptide for 1 h by flow cytometry. Green fluorescence (due to SYTO9 staining) with an excitation/emission wavelength at 485 nm/498 nm was used to demonstrate viable cells, whereas red fluorescence (due to propidium iodide staining) with an excitation/emission wavelength at 535 nm/615 nm was used to show dead cells. Experiments were carried out with three biological replicates and all data were given as mean ± SD, with one-way ANONA was used to determine statistical significance (*P < 0.05, **P < 0.01, ***P < 0.001, ****P < 0.0001).

In view of the strong antibacterial activity of LI14 peptide, we intend to investigate its molecular mechanisms. First, in consideration of Mg^2+^, a stabilizer of the outer membrane, has an obvious influence on the activity of LI14, we speculated that LI14 may target bacterial membrane^32–34^. To test this hypothesis, we first determined the surface morphologies and internal alterations in bacteria cells following treatment by LI14 by scanning electron microscopy (SEM) and transmission electron microscopy (TEM). As demonstrated in Fig. 3a, compared with the intact surface of the control bacteria, substantial bacterial cytolysis in MRSA T144 and distinct membrane damage, such as membrane rupture and pore formation on the surface of *E. coli* B2 were observed. Furthermore, we found remarkable leakage of intracellular contents or concentrated cytoplasm after treatment with LI14 by TEM analysis. Next, considering that LI14 induced evident damage to the membrane of MRSA T144 and *E. coli* B2, we wonder whether LI14 targets certain bacterial membrane components. To this goal, the antibacterial activity changes of LI14 peptide in the presence of exogenous lipopolysaccharide (LPS) or phospholipids were determined. Interestingly, the addition of LPS, phosphatidylglycerol (PG) and cardiolipin (CL) drastically weakened the antibacterial activity of LI14 against MRSA T144 and *E. coli* B2 in a manner of dose-dependent (Fig. 3b–d), implying that LI14 can specifically bind to the LPS located on the bacterial outer membrane and bacterial-specific phospholipids in the inner membrane, thereby inducing the membrane disruption. Subsequently, a hydrophobic fluorescent probe 1*-N-*phenylnaphthylamine (NPN) was utilized to assess the impact of LI14 peptide on the outer membrane permeability in *E. coli* B2. Results showed that LI14 dose-dependently increased the outer membrane permeability of *E. coli* B2 (Fig. 3e). Moreover, the propidium iodide (PI) assay revealed that LI14 also increased inner membrane permeability both in MRSA T144 and *E. coli* B2 (Fig. 3f, g), indicating remarkable damage to the bacterial membrane. Consistently, flow cytometry analysis further demonstrated that LI14 dose-dependently induced the damage of bacteria cell membrane as PI is a nucleic acid-type dye that only crosses damaged cell membranes and binds to DNA to increase red fluorescence^35^ (Fig. 3h). All these findings suggest that LI14 disrupts membrane integrity by targeting bacterial cell membrane-specific components.

### LI14 peptide dissipates the ΔΨ component of the proton motive force

**Fig. 4:**
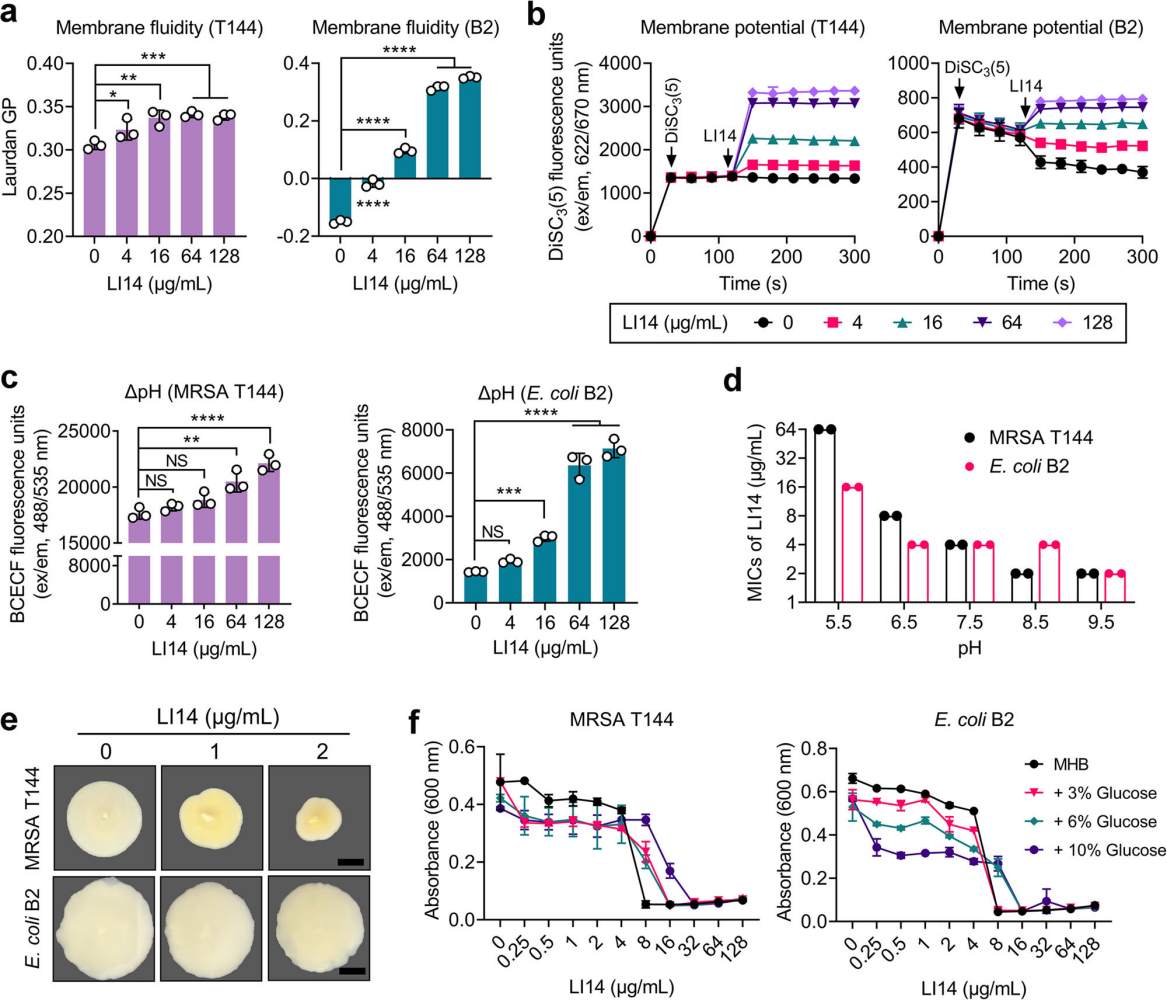
LI14 peptide dissipates the PMF and boosts the production of ROS. **a** Membrane fluidity changes in MRSA T144 and *E. coli* B2 under exposure of LI14 peptide. Membrane fluidity was determined using 10 µM Laurdan, and the fluorescence intensities were detected with emission wavelengths of 435 nm and 490 nm upon excitation at 350 nm. **b** LI14 dissipates membrane potential both in MRSA T144 and *E. coli* B2 in a manner of dose-dependent. A fluorescence probe DiSC_3_(5) (final concentration, 0.5 μM) was injected at 30 s followed by self-quenching and stabilization, then varying concentrations of LI14 peptide were added at 120 s. Fluorescence units of DiSC_3_(5) (excitation/emission wavelengths of 622/670 nm) was monitored during 300 s. **c** Effect of LI14 peptide on the ΔpH component of the PMF both in MRSA T144 and *E. coli* B2, determined by a fluorescence probe BCECF-AM (1 μM) and measured with excitation/emission wavelengths of 488/535 nm. **d** The increase of external pH in media decreases the minimum inhibitory concentrations (MICs) of LI14 peptide against MRSA T144 and *E. coli* B2. **e** Sub-inhibitory concentrations of LI14 peptide decreases the swimming motility of two test strains (MRSA T144 and *E. coli* B2). 0.3% agar media was applied to analyze bacterial swimming motility in the presence of LI14 peptide. After incubation for 48 h, the size of the microsphere was measured and photographed. Scar bar, 0.5 cm. **f** The antibacterial property of LI14 peptide towards MRSA T144 and *E. coli* B2 was inhibited by the adding of glucose exogenously. Experiments were performed with biological replicates. Data were showed as mean ± SD and analyzed by one-way ANONA (*P < 0.05, **P < 0.01, ***P < 0.001, ****P < 0.0001). NS not significant.

Insertion of specific membrane disrupting compounds into lipid bilayers usually causes dramatic changes in membrane fluidity, which usually results in loss of membrane protein, leakage of cellular compounds, and bacterial death^36,37^. Thus, we further evaluated the fluidity of MRSA T144 and *E. coli* B2 under exposure to LI14 peptide by using a membrane-sensitive dye Laurdan. Results showed that LI14 induced a significant increase in Laurdan GP in a manner of concentration-dependent, indicating the decreased membrane fluidity in the presence of LI14 (Fig. 4a). The changes in membrane rigidity would affect bacterial homeostasis, thereby resulting in fundamental metabolic disorders such as the dissipation of proton motive force (PMF).

PMF is essential for the survival of bacteria and is produced by bacterial transmembrane potential, which is composed of the electric potential (ΔΨ) and the transmembrane proton gradient (ΔpH) together^38^. We first assessed the impact of LI14 peptide on the membrane potential using a fluorescent dye DiSC_3_(5), which could accumulate in the cytoplasmic membrane in response to the ΔΨ component of bacterial PMF. When the ΔΨ is disrupted, the probe would be released into the extracellular milieu and resulting in increased fluorescence, and the disruption of ΔΨ would be compensated by increasing ΔpH. Our results showed that DiSC_3_(5) fluorescence intensity was remarkably increased in MRSA T144 and *E. coli* B2 after treatment with the increasing concentrations of LI14 peptide, indicating that LI14 disrupted the ΔΨ component of the PMF (Fig. 4b). Moreover, we selected another fluorescence probe BCECF-AM to evaluate the influence of LI14 on the ΔpH. Interestingly, we found that LI14 induced distinctly increased fluorescence tensity in the tested strains, which is more evident in *E. coli* B2, indicating that LI14 upregulated the ΔpH (Fig. 4c). To prove the above findings, we determined the antibacterial activity of LI14 in different pH conditions ranging from 5.5 to 9.5. Consistent with the above findings, LI14 displayed improved antibacterial activity under alkaline conditions (Fig. 4d). As PMF is composed of ΔΨ and ΔpH, we next analyzed the ultimate effect of LI14 on PMF by swimming motility test. As shown in Fig. 4e, LI14 markedly inhibited bacterial motility at subinhibitory concentrations, indicating the disruption of PMF^39,40^. Consistently, the addition of exogenous glucose partially abolished the antibacterial activity of LI14 peptide, because glucose could supplement PMF (Fig. 4f). These findings indicate that the ΔΨ component of PMF is a potential target of LI14 peptide.

### Bacterial metabolism perturbation contributes to the bactericidal activity of LI14 peptide

**Fig. 5:**
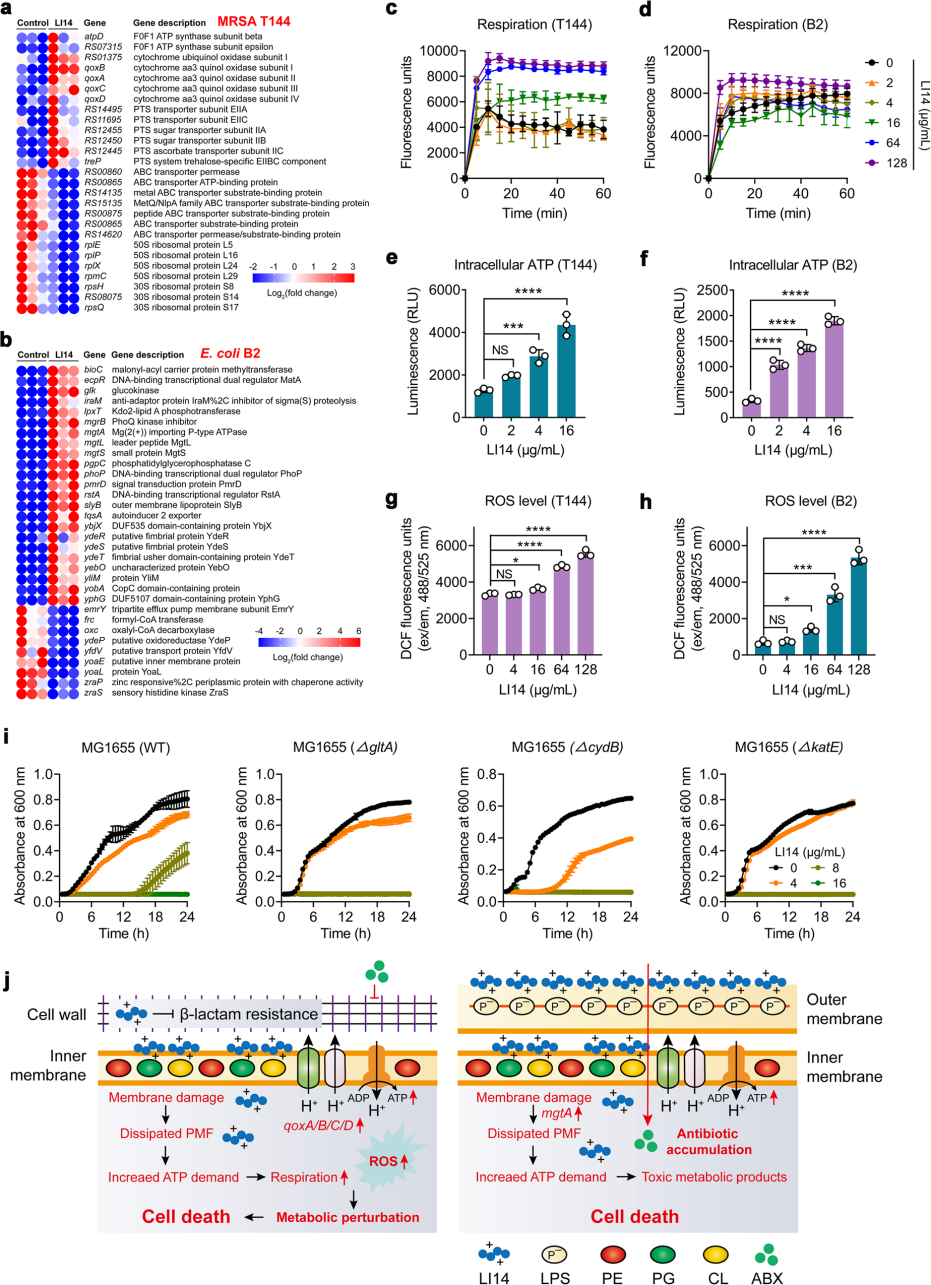
Bacterial metabolism participates in the lethality of LI14 peptide. **a**, **b** The representative differentially expressed genes (DEGs) with larger expression changes in MRSA T144 (**a**) and *E. coli* B2 (**b**) after exposure to LI14 peptide. **c**, **d** LI14 peptide promotes the respiration level of MRSA T144 (**c**) and *E. coli* B2 (**d**). Bacterial respiration was evaluated by resazurin (0.1 µg/mL) and monitored during 60 min with excitation and emission wavelengths of 550 and 590 nm. **e**, **f** LI14 peptide enhances the intracellular ATP production in MRSA T144 (**e**) and *E. coli* B2 (**f**). Using an Enhanced ATP Assay Kit, the intracellular ATP levels of MRSA T144 and *E. coli* B2 were measured by monitoring the corresponding luminescence signals. **g**, **h** LI14 peptide triggers the production of ROS both in MRSA T144 (**g**) and *E. coli* B2 (**h**). 2’,7’-Dichlorodihydrofluorescein diacetate (DCFH-DA, 10 µM) was used to determine the levels of ROS in bacteria after exposure to LI14 peptide for 1 h (excitation/emission wavelengths of 488/525 nm). Experiments in **e**–**h** were conducted with three biological replicates. Data were presented as mean ± SD and analyzed by one-way ANONA (*P < 0.05, ***P < 0.001, ****P < 0.0001). NS, not significant. **i** Growth curve assays of *E. coli* MG1655 and its gene knockout mutants (*ΔgltA*, *ΔcydB,* and *ΔkatE*) in the presence of increasing concentrations of LI14 peptide. **j** Mechanism of actions accounting for the antibacterial activity of LI14 peptide in Gram-positive (left) and Gram-negative bacteria (right), respectively.

We next performed transcription analyses of MRSA T144 and *E. coli* B2 to acquire a better understanding of the underlying molecular mechanisms of the LI14 peptide. It revealed that there were 231 differentially expressed genes (DEGs) in MRSA T144 and 165 DEGs in *E. coli* B2 (Supplementary Fig. 6). The representative DEGs with larger expression changes were presented in Fig. 5a and b. We found that these upregulated DEGs were associated with energy metabolism and oxidative phosphorylation, while ribosomal protein-related genes were substantially downregulated by LI14 in MRSA T144 (Fig. 5a). In addition, DEGs associated with membrane damage (e.g., *mgtA*, *mgtL*, and *mgtS*) were substantially upregulated in *E. coli* B2 (Fig. 5b), which were consistent with our previous findings (Fig. 3). To verify the transcriptome results, we first analyzed the changes in bacterial respiration levels after LI14 treatment using the oxygen-sensitive dye resazurin. In agreement with transcriptome results, we found that LI14 distinctly improved bacterial respiration levels in MRSA T144, while there was no significant change in *E. coli* B2 (Fig. 5c, d). A dose-dependently increase of intracellular ATP levels in bacteria after exposure to LI14 was also found (Fig. 5e, f). These findings are supported by a recent study, which reported that the efficiency of bactericidal agents is usually linked to bacterial cellular respiration and the over-production of ATP levels^41^. Accelerated respiration and enhanced metabolism will inevitably lead to the accumulation of toxic products, so we further analyzed the production of ROS, which is an important indicator of bactericidal antibiotics^42,43^. Like many bactericidal antibiotics, LI14 dose-dependently promoted the accumulation of intracellular ROS (Fig. 5g, h), thereby exacerbating membrane damage. By contrast, the supplementation of ROS scavenger *N*-acetyl-L-cysteine (NAC)^44^ drastically abolished the antibacterial activity of LI14, indicating that the formation of ROS by LI14 is an important factor for its bactericidal activity (Supplementary Fig. 7). To further verify these findings, we conducted gene knockout on metabolism-related genes using a reference strain *E. coli* MG1655 that is easily genetically manipulated. Interestingly, we found that the citrate synthase (*gltA*)-deficient and cytochrome bd-I ubiquinol oxidase subunit II (*cydB*)-deficient MG1655 displayed increased susceptibility to LI14 peptide (Fig. 5i), implying that the antibacterial activity of LI14 is associated with bacterial metabolism. In addition, the deletion of *katE*, an antioxidant gene required for catalase activity (Fig. 5i), improved the inhibitory effect of LI14, highlighting the important role of oxidative damage. Taken together, our results suggest that LI14 peptide triggers membrane damage, dissipates PMF, and perturbs bacterial metabolism, thereby resulting in the accumulation of toxic products and bacterial death (Fig. 5j).

### LI14 peptide sensitizes clinically relevant antibiotics and is efficacious in vivo

Recently, combination therapy, especially antibiotic adjuvants, has been widely studied to alleviate the crisis of antibiotic resistance^45–47^. We evaluated the potential synergistic activity of LI14 peptide with a set of clinically relevant antibiotics. Interestingly, we found that LI14 effectively potentiated multi-classes of antibiotics including ciprofloxacin, doxycycline, vancomycin, rifampicin against MDR *E. coli* B2 (FICI, 0.078 to 0.25), and tigecycline against *tet*(X4)-positive *E. coli* B3-1 with a FICI of 0.125 (Supplementary Fig. 8 and Supplementary Table 5). Similar potentiating effects were also observed in sensitive *E. coli* ATCC 25922 (Supplementary Fig. 9 and Supplementary Table 6). For notorious MRSA T144, LI14 peptide remarkably enhanced the activity of ampicillin and doxycycline with a FICI of 0.078 and 0.188, respectively (Supplementary Fig. 10 and Supplementary Table 7). The strong synergistic effect with ampicillin was consistent with transcriptome results, which showed that LI14 supplementation markedly reduced beta-lactam resistance (Supplementary Fig. 6). These results highlight the potency of LI14 as a novel broad-spectrum antibiotic synergist.

**Fig. 6:**
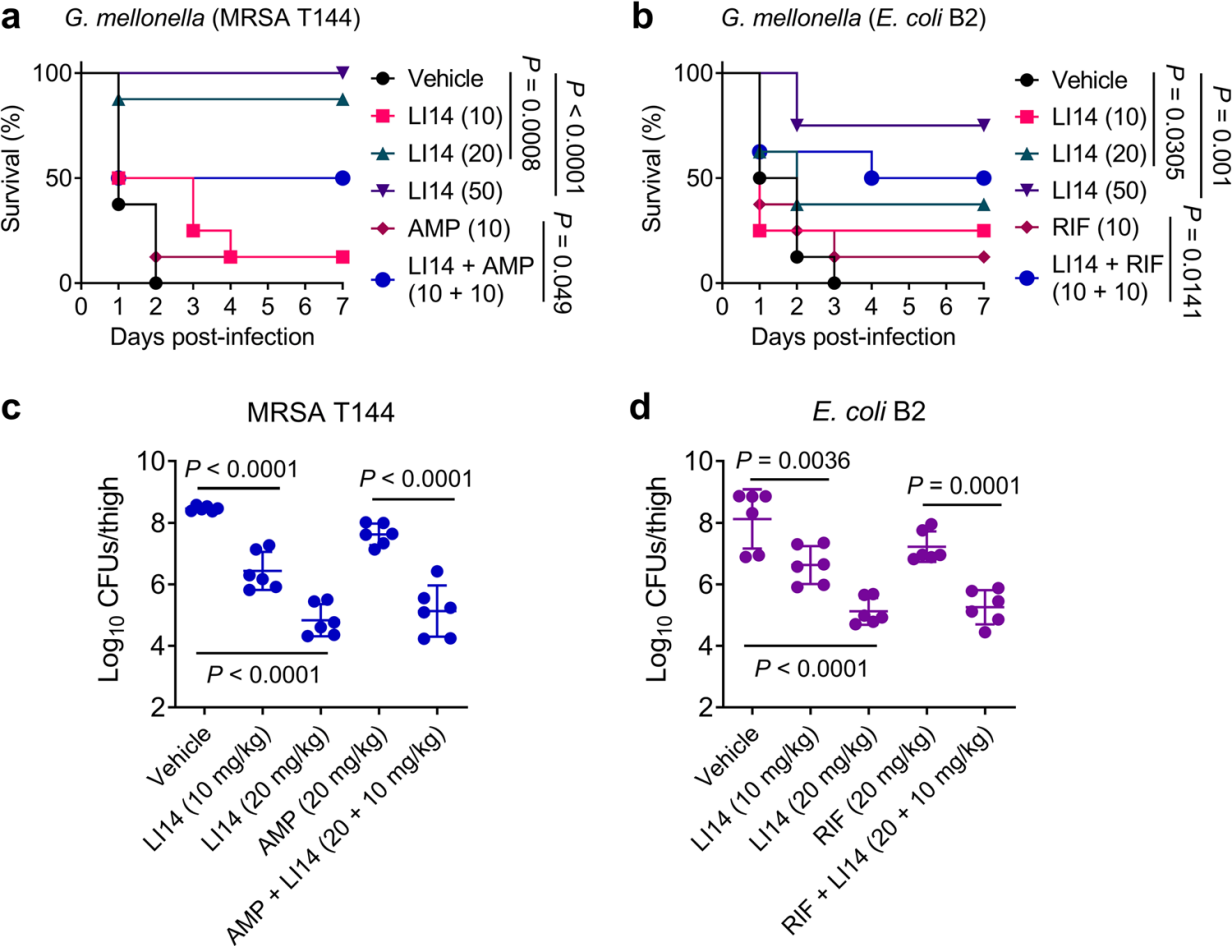
Therapeutic potential of LI14 peptide in animal models of infection. **a**, **b** LI14 alone or in combination with ampicillin (AMP)/rifampicin (RIF) improved the survival of *G. mellonella* infected with MRSA T144 (**a**) or *E. coli* B2 (**b**). *G. mellonella* (*n* = 8 biologically independent animals per group) were first infected by two test strains, respectively, and then treated with different concentrations of LI14 peptide or their combination at 1 h after infection. Survival rate of larvae was monitored during 7 days. *P* values were calculated using log-rank (Mantel-Cox) test. **c**, **d** LI14 peptide alone or in combination with ampicillin/rifampicin reduced bacterial burden in a neutropenic mouse thigh infection model. Neutropenic CD-1 mice (*n* = 6 biologically independent animals per group) were administered a non-lethal dosage of MRSA T144 (**c**) or *E. coli* B2 (**d**, 10^5^ CFUs) intramuscularly, and then treated with a standard injection of LI14 peptide (10 or 20 mg/kg), ampicillin or rifampicin (20 mg/kg), or their combination at 1 h after nfection. The amount of bacteria load in the thighs of mice after 48 h treatment was determined. The Mann–Whitney U-test was used to evaluate the *P* values for the data, which were given as mean ± SD.

Considering that the LI14 peptide exhibited potent antibacterial and synergistic activity in vitro, we next evaluated the in vivo efficacy of LI14 alone or in combination with ampicillin/rifampicin in various animal infection models. In the *Galleria mellonella* infection model, the larvae in the control group all died during 48–72 h, while the survival rate of the larvae was significantly improved with the increasing doses of LI14 (Fig. 6a, b). In particular, the survival rate of larvae was 100% and 75%, respectively, in MRSA T144 and *E. coli* B2 infected groups at 50 mg/kg of LI14. Moreover, compared with the antibiotic administration alone group, the survival of larvae in combination groups was remarkably enhanced to 50% in two bacterial infected groups. Next, we verified the in vivo efficacy of LI14 by constructing a neutropenic mouse thigh infection model infected with MRSA T144 or *E. coli* B2^48^. Encouragingly, in both MRSA T144 and *E. coli* B2 infected mice, we observed that LI14 significantly decreased bacterial loads in the thigh of mice, with ~2-log_10_ at 10 mg/kg and 4-log_10_ at 20 mg/kg reductions in CFUs compared with vehicles (Fig. 6c, d). Also, the combinations of LI14 with ampicillin or rifampicin exhibited more potent CFUs reduction of two pathogens compared with the antibiotic alone group. Last, the efficacy of LI14 was assessed using an ex vivo wounded mouse skin infection model. As shown in Supplementary Fig. 11, either MRSA T144 or *E. coli* B2 induced infections, LI14 all distinctly improved wound closure and decreased the number of colonies in a dose-dependent manner during 8 days. Furthermore, H.E. staining showed that the granulation tissues were much thicker in the LI14 or combination group, and LI14 also attenuated the congestion and inflammatory cell infiltration, indicating that LI14 may decrease the systemic inflammatory response induced by local infection. Together, these results indicate that LI14 peptide is highly effective against in vivo bacterial infections caused by MDR pathogens.

## Discussion

Antibiotics, as one of the highly successful drugs, have saved millions of lives in the past decades. However, due to their long-term and extensive use, there is a rapid rise in bacterial resistance to existing antibiotics, especially for the Gram-negative members of the ESKEPE panel. There is an urgent need for the development of new antibiotic candidates with new scaffolds, unique mechanisms of action, and low propensity to develop resistance. Here, we reported a panel of new peptide compounds with heptapeptide repeat sequence and its derivatives. Interestingly, we found that LI14 with a novel lead structure was highly effective against all tested MDR Gram-positive and Gram-negative pathogens, including MRSA and *E. coli* B2 co-harboring *mcr-1* and *bla*_NDM-5_, implying the broad-spectrum bactericidal activity of LI14. Moreover, LI14 showed tolerance against high concentrations of serum and pepsin, and displayed low toxicity both in vitro and in vivo. Mechanistic studies indicated that LI14 was a bacterial membrane-targeted compound, which induced membrane damage, dissipated membrane potential and led to bacterial metabolism perturbation. Most importantly, LI14 peptide was efficacious in multiple animal models of infections caused by MDR pathogens, indicating that LI14 is a promising candidate for further development as a novel antibiotic.

Amphiphilicity, ɑ-helicity and appropriate peptide chain length are key factors for AMP activity^49^. In our study, LI7 showed very weak antibacterial activity against most of tested strains, which may be due to the short length of peptide chains that can not to form a secondary structure. Compared with LI7 and LI21, LI14 exhibited the best antibacterial activity. Furthermore, LI14-A_6_ was modified by substituting Arg with Ala at position 6, and had a lower ɑ-helicity proportion and displayed no antibacterial activity, which was consistent with the previous notion that ɑ-helicity is one of the prerequisites for the activity of AMPs^50^. Moreover, stability and safety are also the prerequisites for the application of AMPs^51,52^. Our data showed that LI14 was still effective under different physiological conditions, such as acid and alkali conditions, serum and pepsin, and had no detectable toxicity. This is in stark contrast to some previously reported peptide antibiotics, for example, LL-37 displayed strong activity in MHB broth medium whereas serum fully abolished its activity with a greater than 128-fold change of MIC value^28^.

Biofilms and persister cells are important causes of chronic and recurrent infections^53,54^, yet are usually overlooked in the screening of new antibiotics. We found that LI14 was able to inhibit the formation of biofilms and eliminate the mature biofilms. Furthermore, persister cells are usually in a dormant status and highly resistant to antibiotics, which poses a serious challenge to antibiotic therapy. It was reported that acyldepsipeptide 4 (ADEP4) could effectively eliminate persister cells but along with rapid resistance development in *S. aureus*^55^. Here, we showed that LI14 had an above 99% clearance rate against MRSA T144 and *E. coli* B2 persisters within 2 h.

Screening of novel antibacterial agents that target bacterial membranes is a very promising strategy, as membrane-targeting antibiotics usually have rapid bactericidal activity^56,57^ and have less prone to develop antibiotic resistance as bacterial membrane can hardly spontaneous mutation without loss of function^58^. For example, the broad-spectrum adjuvant SLAP-S25 exhibited synergistic activity by targeting the PG in the membrane^30^. In our study, we found that LI14 could target the LPS of the outer membrane and PG of the inner membrane, and finally induce membrane damage. The damage of biophysical integrity by LI14 was accompanied by a series of physiological changes, including increased membrane permeability, disrupted PMF, bacterial metabolic disorder and the leakage of cytoplasmic contents. It is noteworthy that the dissipation of PMF plays an important role in the lethality of LI14, which impairs the basic functions of bacteria and accelerates its death. A new broad-spectrum antibiotic termed halicin was recently identified utilizing a deep learning method, which selectively disrupts the ΔpH component of the PMF^26^. Furthermore, as potential modulators of PMF, compounds I1-I3 and D1-D3 showed a lethal effect against MRSA T144 by inhibiting electron transport and ATP production^38^. In our study, we found that LI14 dissipated the ΔΨ component of PMF in both Gram-positive and Gram-negative bacteria. These findings underscore that bacteria PMF can serve as a potential target for the development of novel antimicrobial agents. Furthermore, LI14 resulted in metabolic perturbations, increased ATP levels and the accumulation of toxic products. There is growing evidence that bacterial metabolism is linked to antibiotic efficacy^10,59^. For instance, a recent study revealed that stress-induced changes in ATP utilization and demand, as a homeostatic response, severely drive lethal metabolic alterations^60^.

In conclusion, we report that LI14 peptide is an excellent membrane-targeted antibiotic candidate to combat recalcitrant infections elicited by MDR pathogens because of its broad-spectrum antibacterial, anti-biofilms and persisters activity; its low resistance development and toxicity, high stability, as well as its systemic efficacy in animal models of infection. In addition, our study highlights the importance of PMF disruption and metabolic perturbation, providing new insights into the mechanisms of action of AMPs.

## Methods

### Peptide synthesis and validation

In this study, solid-phase methods were used to synthesize the engineered peptides by GL Biochem (Shanghai, China). We determined the molecular weights of peptides using matrix-assisted laser desorption/ionization time-of-flight mass spectrometry (MALDI-TOF MS). In addition, reversed-phase high-performance liquid chromatography (RP-HPLC) was used to assess peptide purity and retention time. Using the HeliQuest website (www.heliquest.ipmc.cnrs.fr/cgi-bin/ComputParamsV2.py), the hydrophobic moment and charge of all peptides were calculated.

### Bacterial strains and chemical reagents

Supplementary Table 1 contains a list of all strains used in this study. By using suicide plasmid pLP12 and homologous recombination, deletion mutants of *Escherichia coli* MG1655 were obtained followed by PCR confirmation. The bacterial strains were cultured on Mueller-Hinton Broth (MHB) or Mueller-Hinton Agar plates (MHA) at 37 °C, unless otherwise noted. The antibiotics used in this study were obtained from China Institute of Veterinary Drug Control. Other chemical compounds were obtained from TCI. (Shanghai, China).

### Animal studies and ethical statement

We obtained female CD-1 mice (aged 8 weeks; 20–25 g) and rats (6–8-week-old) from Comparative Medicine Center of Yangzhou University. Prior to infection studies, all the mice were fed under standardized environmental conditions for one week. In this research, all animal experiments were carried out according to the guidelines of Jiangsu Laboratory Animal Welfare and Ethical of Jiangsu Administrative Committee of Laboratory Animals. Each animal study was approved by Jiangsu Laboratory Animals Administrative Committee, and all protocols were accompanied by a permission number (SYXKSU-2007-0005). The laboratory animal usage license number is SCXK-2017-0044, certified by the Jiangsu Association for Science and Technology.

### Antibacterial and bactericidal activity tests

#### Broth microdilution assay

An antimicrobial activity study was conducted on all peptides against a panel of laboratory bacteria, including ATCC source and clinical isolates with resistance determinants. According to CLSI2021 guidelines, we tested the minimum inhibitory concentrations (MICs) of peptides using standard broth micro-dilutions. Briefly, bacteria grown to exponential phase were diluted in 1:1000 to MHB, subsequently, equivalent amounts of bacterial suspension (1.5 × 10^6^ CFUs/mL) were mixed with different concentrations of peptides in a sterilized 96-well microtiter plate (Corning). MIC values were calculated after 16 to 18 h of incubation at 37 °C based on the lowest concentrations of peptides that did not show visible bacterial growth^37^.

#### Checkerboard assays

Through checkerboard assay using two-fold serially diluted drugs (8 × 8 matrix), we evaluated the synergistic effects of LI14 with different mechanisms of antibiotics. The absorbance of cultures at 600 nm was determined by a Microplate reader after 18 h of incubation with (1.5 × 10^6^ CFUs/mL) bacterial suspension. The FIC index (FICI) was calculated by performing two biological replicates for each combination. The formula is as follows: FIC index = FICIa + FICIb = MIC_ab_/MIC_a_ + MIC_ba_/M_b_. An FIC index of ≤0.5 indicates synergy^48^.

#### Time-killing assay

Bacteria from the exponential phase were diluted into MHB at 1:1,000, and bacteria suspensions (~10^5^ CFUs/mL for Gram-positive bacteria, ~10^6^ CFUs/mL for Gram-negative bacteria) were mixed with various concentrations of LI14 peptide and incubated at 37 °C for 0.5, 1, 2, 4, and 12 h, respectively^26^. At intervals, samples were serially diluted ten-fold and plated on MHA plates. The amount of primary CFUs/mL was determined after overnight incubation of bacterial colonies.

#### Prevention of biofilm formation

LI14 peptide was tested for its effect on biofilm formation as previously described^61^. Briefly, 10^6^ CFUs/mL bacteria suspension was incubated with sub-MIC concentrations of LI14 (0–4 μg/mL) for 36 h under 37 °C. Next, planktonic bacteria were removed, and biofilms were first fixed with methanol for 15 min and then stained with 0.1% crystal violet for 15 min, followed by washing, and 33% acetic acid was applied to solubilize the biofilms. At 570 nm, the absorbance of cell cultures was measured to determine biofilm creation.

#### Treatment of established biofilms

In MHB medium, a mid-logarithmic growth-phase culture was diluted to 10^6^ CFUs/ml. A 96-well flat bottom plate was filled with 100 μL of this bacterial suspension per well, which was cultured at 37 °C for 36 h to create mature biofilms^62^. The planktonic bacteria were then discarded, and the remaining biofilm cells were cultured at 37 °C for 2 h with escalating doses of LI14 (0–128 μg/mL). Plates were sonicated for 10 min after incubation to remove adhering bacteria, and the number of live bacteria was counted microbiologically.

#### Bactericidal activity against persisters

Briefly, the bacteria suspensions after washing were exposed to 50-fold MIC concentrations of vancomycin and tigecycline, respectively, and co-incubation for 4 h to obtain persister cells^61^. After the removal of planktonic bacteria, the bacterial solution was exposed to different concentrations of LI14 peptide (0–128 μg/mL) for another 6 h incubation. Subsequently, the primary CFUs/mL were calculated.

#### Resistance development study

The ability of LI14 peptide to induce resistance was studied as previously described^63^. Resistance to the therapeutically important drugs rifampicin and ciprofloxacin was measured using *S. aureus* ATCC 29213 and *E. coli* ATCC 25922 as test strains. The middle and final MICs were monitored after eighty such serial passages exposed to the tested medicines (0.5-fold MIC), and the fold change in MIC increase was recorded.

### Stability analysis

#### Salt and serum stability

MRSA T144 and *E. coli* B2 in the exponential growth phase were incubated with LI14 peptide supplemented with 10 mM Na^+^, K^+^, Mg^2+^, 10% Dulbecco’s modified Eagle medium (DMEM), and 10% fetal bovine serum (FBS) for 16–18 h. The stability of LI14 was evaluated by the change of its antibacterial activity under different conditions.

#### Thermal, pH, and proteolytic stability

LI14 were preincubated for 1 h at various temperatures (40–121 °C), pH (2–12), and proteases (pepsin, trypsin, and papain, final concentration 1 mg/mL). After pH treatment, samples were adjusted to pH = 7.2 and MIC assays were used to detect the residual antibacterial activity. Following the incubation with the proteases, the leftover protease was precipitated with acetonitrile and removed by centrifugation at 3000 × *g*, followed by a MIC test.

### Safety evaluation

#### Hemolysis activity

The hemolytic rate of LI14 peptide was examined using sterile defibrated sheep erythrocytes in line with a preceding study^40^. Simply speaking, 8% sheep erythrocytes were prepared and incubated with serial-concentration of LI14 peptide (0–128 μg/mL) under 37 °C for 1 h, sterile PBS serving as blank control and ddH_2_O serving as a positive control. After incubation, an Infinite M200 Microplate reader (Tecan) was used to detect the absorbance of the supernatant at 576 nm, and the hemolysis rate was estimated based on the controls.

#### Acute toxicity in mice

LI14 (10 mg/kg, body weight) was intra-peritoneally injected into CD-1 mice (*n* = 4 biologically independent animals per group) every day for 6 days^64^. Mice were weighed daily and the behaviors of the mice were inspected for one week. Blood from mice was collected on the seventh day for whole-blood cell analysis and kidney chemistry profile. At the same time, the heart, liver, spleen, lung, and kidney of mice were collected for H.E. staining.

### CD spectrum assay

Peptides at a final concentration of 0.1 mg/mL were respectively dissolved in 0.01 M PBS (pH = 7.2), 50 μM LPS, 50 mM SDS, and 50% trifluoroethanol (TFE). J-810 spectropolarimeter (Jasco, Tokyo, Japan) was used to measure CD values with a spectrum of from 190 to 300 nm at 25 °C.

### Antibacterial mechanisms experiment

#### SEM and TEM assay

The surface morphology alterations of bacterial cells treated by LI14 peptide were evaluated by scanning electron microscope (SEM). Briefly, overnight cultured bacteria suspensions were cleaned twice and incubated with LI14 peptide (10-fold MIC) at 37 °C for 1 h. The cell pellets were extracted after incubation, washed twice in PBS, and fixed overnight at 4 °C with 2.5% glutaraldehyde. Next, samples were dehydrated by gradient ethanol (30%, 50%, 70%, 90%, and 100%)^25^. The specimens were then dried, gold-coated, and detected with GeminiSEM 300 (ZEISS, Germany).

The morphological and intracellular changes of bacterial cells following LI14 treatment were examined using a transmission electron microscope (TEM).

#### Phospholipid inhibition analysis

The impact of exogenous LPS and various kinds of lipids on the activity of LI14 peptide were assessed by the checkerboard microdilution assay. Briefly, lipopolysaccharide (0–128 μg/mL), phosphatidylglycerol (0–16 μg/mL), phosphatidylethanolamine (0–16 μg/mL), cardiolipin (0–16 μg/mL), phosphatidylcholine (0–16 μg/mL) and LI14 peptide were co-incubation with bacterial suspensions in 96-well plate. After 16–18 h incubation, to assay the MICs of LI14 in the presence of LPS and different lipids were recorded.

#### Outer membrane and cell membrane permeability

The permeability of the bacterial outer membrane and cell membrane was measured using the fluorescent dyes 1-*N*-phenylnaphthylamine (NPN) and propidium iodide (PI), respectively. Briefly, final concentrations of NPN (10 μM) and PI (5 μM) were incubated with bacterial suspensions for 30 min, subsequently, treated by LI14 (0–128 μg/mL) for 1 h. Then the outer/cell membrane permeability was then measured using an Infinite M200 Microplate reader (Tecan) with excitation/emission wavelengths of 350 nm/420 nm (NPN) and 535 nm/615 nm (PI).

#### Flow cytometry assay

Bacterial suspensions (10^6^ CFUs/mL) were incubated with LI14 peptide (0–64 μg/mL) for 1 h at 37 °C. Then, PI (5 mM, 3 μL) and SYTO 9 (0.835 mM, 3 μL) were added to the mixture of bacteria and LI14 peptide and incubated for 15 min at room temperature in dark. About 100,000 ungated events were measured by CytExpert Flow Cytometer (Beckman, USA) and analyzed by CytExpert 2.0 software (Beckman, USA)^35^.

#### Membrane fluidity assay

The exponential bacterial suspensions were centrifuged and washed with PBS, then bacterial suspensions were incubated with 10 µM Laurdan in dark for 30 min at 37 °C. The stained cell cultures were washed with PBS two times and concentrated. Next, different concentrations of LI14 were mixed with the stained bacterial cells to incubate for another 1 h at 37 °C. The Laurdan fluorescence levels were measured using a Microplate reader (Tecan, Männedorf, Switzerland) with emission wavelengths of 435 nm and 490 nm upon excitation at 350 nm after 1 h incubation in the dark. GP = (I_435_ – I_490_)/(I_435_ + I_490_)^37^ was used to determine the Laurdan GP. All experiments were conducted in biological triplicate.

#### Cytoplasmic membrane potential

The changes of membrane potential treated by LI14 were evaluated using 3,3’-dipropylthiadicarbocyanine iodide (DiSC_3_(5), 0.5 μM)^65^. Briefly, bacterial samples were first cultured for 30 min with (DiSC_3_(5), 0.5 μM) before being treated for 1 h with various doses of LI14 peptide. The excitation wavelength of 622 nm and the emission wavelength of 670 nm was used to detect the bacteria’s dissipated membrane potential.

#### ΔpH measurement

The exponential bacterial suspensions were centrifuged and washed with PBS, then bacterial suspensions were incubated with a pH-sensitive fluorescence probe BCECF-AM (1 µM)^66^ in dark for 30 min at 37 °C. Following treated with different concentrations of LI14 peptide for 1 h at 37 °C. The excitation/emission wavelength of 488 nm/535 nm was detected to calculate the fluorescence value.

#### Swimming motility experiment

0.3% (w/v) agar media containing trypticase peptone (10 g/L), NaCl (10 g/L), and yeast extract (5 g/L) was used to evaluate bacterial swimming motility. Wait until the temperature drops to 50 °C, subinhibitory concentrations of LI14 were added (final concentrations, 0, 1, and 2 μg/mL). In the center of each plate, a volume of 2 μL of bacterial suspensions (10^6^ CFUs/mL) was deposited and allowed it to dry. The swimming motility of the bacteria was evaluated by measuring the microsphere diameter after incubation at 37 °C for 48 h.

#### Transcriptomic analysis

Early-exponential bacteria (MRSA T144 and *E. coli* B2) were treated by LI14 peptide (8-fold MIC) for 4 h at 37 °C. The total RNA of samples was extracted using an EASYspin Plus kit (Aidlab, Beijing, China), quantified with a Nanodrop spectrophotometer (Thermo Scientific, MA, USA), and sequenced using the Illumina Hiseq 2000 system (Majorbio, Shanghai, China) after incubation.

#### Bacterial respiration analysis

The influence of LI14 peptide on bacterial respiration was evaluated by oxygen-sensitive dye resazurin^67^. In brief, the exponential bacterial suspensions were centrifuged and washed with PBS, then added different concentrations of LI14 peptide (0–128 µg/mL) and constant resazurin (0.1 µg/mL), then dynamically monitored fluorescence change for 1 h with excitation/emission wavelength of 550/590 using the Infinite M200 Microplate reader (Tecan).

#### Intracellular ATP determination

Using an Enhanced ATP Assay Kit (Beyotime, Shanghai), the intracellular ATP concentrations of MRSA T144 and *E. coli* B2 were measured. Briefly, the exponential bacterial suspensions were incubated with LI14 peptide ranging from 0 to 16 µg/mL for 1 h. Bacterial centrifugal cleaning and the bacterial pellet were collected. Then, lysis solution was added to lysate bacterial cells. Using the Infinite M200 Microplate reader (Tecan), intracellular ATP levels were calculated from luminescence signals.

#### ROS measurements

The amounts of ROS in MRSA T144 and *E. coli* B2 treated with LI14 peptide were measured using 2’,7’-Dichlorodihydrofluorescein diacetate (DCFH-DA, 10 µM). Briefly, bacteria were firstly incubated with a fluorescent probe DCFH-DA (10 µM) at 37 °C for 30 min and then washed with PBS to reduce the influence of extracellular fluorescence, next probed cells were treated with LI14 peptide (0–128 µg/mL) for 1 h. The levels of ROS were evaluated using a Microplate reader (Tecan, Männedorf, Switzerland) to measure fluorescence intensity (λexcitation = 488 nm, λemission = 525 nm).

#### NAC assay

The significance of ROS in the function of the LI14 peptide was investigated using *N*-acetyl-L-cysteine (NAC). Briefly, NAC (1, 5, and 10 mM) was added to the mixture of LI14 and bacterial culture. The effects of different concentrations of NAC on bacterial growth exposed to LI14 peptide were analyzed by monitoring bacterial growth curves.

### In vivo efficiency

#### Galleria mellonella infection model

*Galleria mellonella* larvae (supplied by Huiyude Biotech Company, Tianjin, China) were separated into six groups (*n* = 8 biologically independent animals per group) and infection models by injecting MRSA T144 and *E. coli* B2 cultures, respectively (10 µL, 1.0 × 10^5^ CFUs per larvae) into the right posterior gastropoda^42,48^. The infected larvae were treated by giving LI14 peptide (10, 20, 50 mg/kg), ampicillin/rifampicin (10 mg/kg) or a mixture of LI14 with ampicillin/rifampicin (10 + 10 mg/kg) in the left posterior gastropoda after 1 h infection, with the PBS injection group serving as a control treatment. Within 7 days, survival rates of *Galleria mellonella* larvae were inspected and recorded.

#### Neutropenic mouse thigh infection model

Female CD-1 mice (*n* = 6 biologically independent animals per group) were first given cyclophosphamide (two consecutive injections of 150 and 100 mg kg^−1^ at 4 and 1 day before infection) to construct neutropenia models. The mice’s right thighs were then injected with 100 μL MRSA T144 or *E. coli* B2 suspensions (10^5^ CFUs per mouse). Post-infection of 1 h, 100 μL LI14 peptide (10 or 20 mg/kg), ampicillin/rifampicin (20 mg/kg) or a combination of LI14 with ampicillin/ rifampicin (10 + 20 mg/kg) were given by intraperitoneal injection. All mice were euthanized 48 h after drug administration, and right thigh muscle tissue was removed aseptically, homogenized, serially diluted, and plated on MHA to qualify bacterial loads after overnight incubation at 37 °C.

#### Rat skin wound infection model

The rats (*n* = 4 biologically independent animals per group) were anesthetized and a 2 cm wound model was constructed on their back. Then, 100 μL of MRSA T144 or *E. coli* B2 cultures were dropped on the wounds (10^6^ CFUs per mouse), respectively. At 1 h post-infection, 100 μL of LI14 peptide (5 or 10 μg), or the combination of LI14 peptide with ampicillin/rifampicin (5 + 5 μg) were gently inoculated over the scar with a pipette tip, respectively. Wound size was recorded for 8 days, and on the 8th day, the rats were euthanized, and wound tissue was removed aseptically to measure CFUs and for following hematoxylin and eosin (H&E) staining.

### Statistics and reproducibility

Statistical analysis was performed using GraphPad Prism version 9.0. All experiments were performed with at least three independent biological replicates and data are presented as mean ± standard deviation (SD). Unless otherwise noted, unpaired *t*-test between two groups or one-way ANOVA among multiple groups were used to calculate *P*-values. Significance levels are presented by numbers of asterisks: *P < 0.05, **P < 0.01, ***P < 0.001, and ****P < 0.0001.

### Reporting summary

Further information on research design is available in the Nature Research Reporting Summary linked to this article.

## Supplementary information

Supplementary information

Description of Additional Supplementary Files

Supplementary Data 1

Reporting Summary

## Data Availability

RNA-sequencing data have been deposited in the National Center for Biotechnology Information (NCBI) Sequence Read Archive (SRA) database (PRJNA825494 and PRJNA825633). Source data for the main figures are provided in Supplementary Data 1. All other data are available from the corresponding authors.
