# Supplementary material for: The antimicrobial peptide LI14 combats multidrug-resistant bacterial infections
Source: Commun Biol. 2022 Sep 7;5:926. doi: 10.1038/s42003-022-03899-4 (PMC9452538; doi:10.1038/s42003-022-03899-4)
Supplement: Supplementary file 1 — Supplementary information [file 42003_2022_3899_MOESM1_ESM.pdf]

Supplementary Information

Content list

Supplementary Figures

Supplementary Fig. 1 --2

Supplementary Fig. 2 -- 3

Supplementary Fig. 3 -- 4

Supplementary Fig. 4 -- 5

Supplementary Fig. 5 -- 6

Supplementary Fig. 6 -- 8

Supplementary Fig. 7 -- 9

Supplementary Fig. 8 --10

Supplementary Fig. 9 --11

Supplementary Fig. 10 -- 12

Supplementary Fig. 11 -- 13

Supplementary Tables

Supplementary Table 1 --15

Supplementary Table 2 --16

Supplementary Table 3 -- 17

Supplementary Table 4 --18

Supplementary Table 5 --19

Supplementary Table 6 --20

Supplementary Table 7 --21

Figures

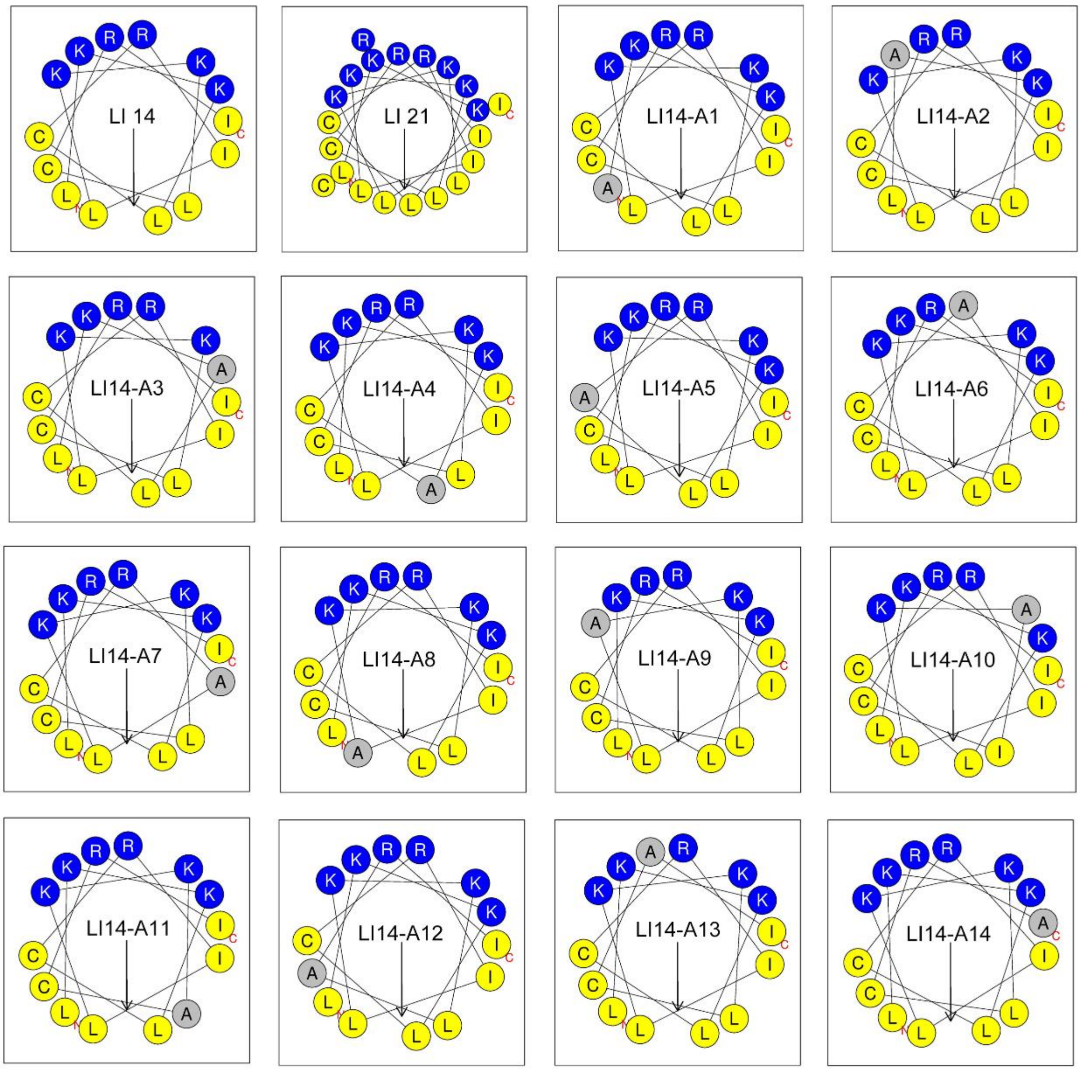

Supplementary Fig. 1 Helical wheel projections of peptides and alanine substitutions of LI14.

Helical wheel projections of peptides. Amino acids in blue are positively charged, while in yellow are hydrophobic.

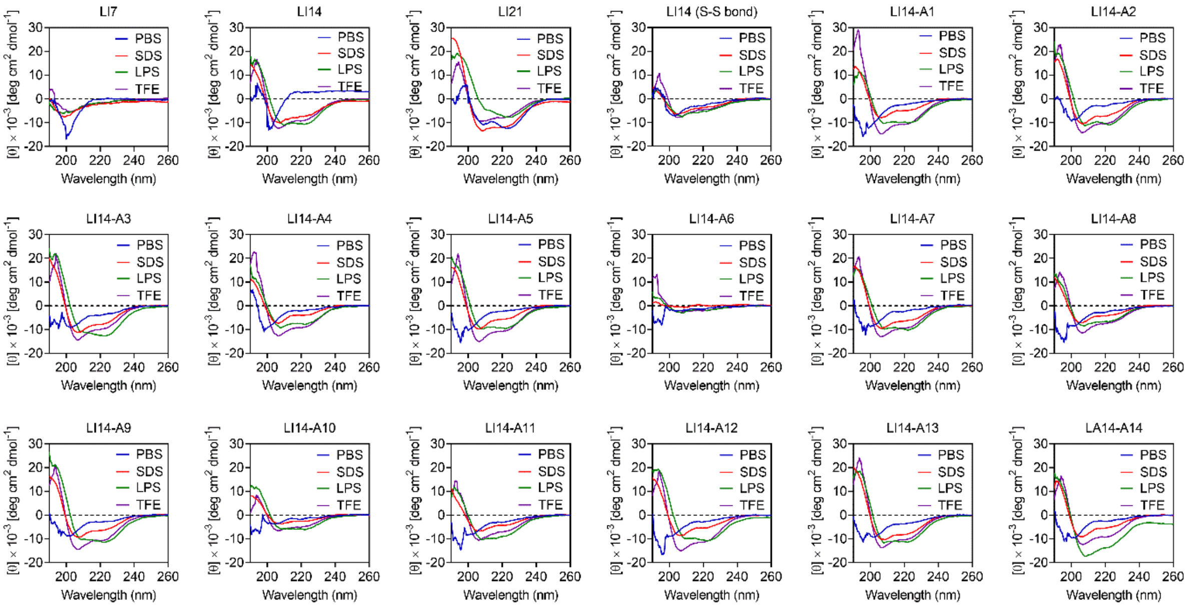

Supplementary Fig. 2 CD spectra of engineered LI peptides.

Engineered peptides were dissolved in 10 mM sodium phosphate buffer (pH = 7.2) (blue), 50 mM SDS (red), 50 μM LPS (green) and 50% TFE (purple). The mean residue ellipticity was plotted against wavelength. The values from three scans were averaged per sample, and the peptide concentrations were fixed at 100 μg/mL.

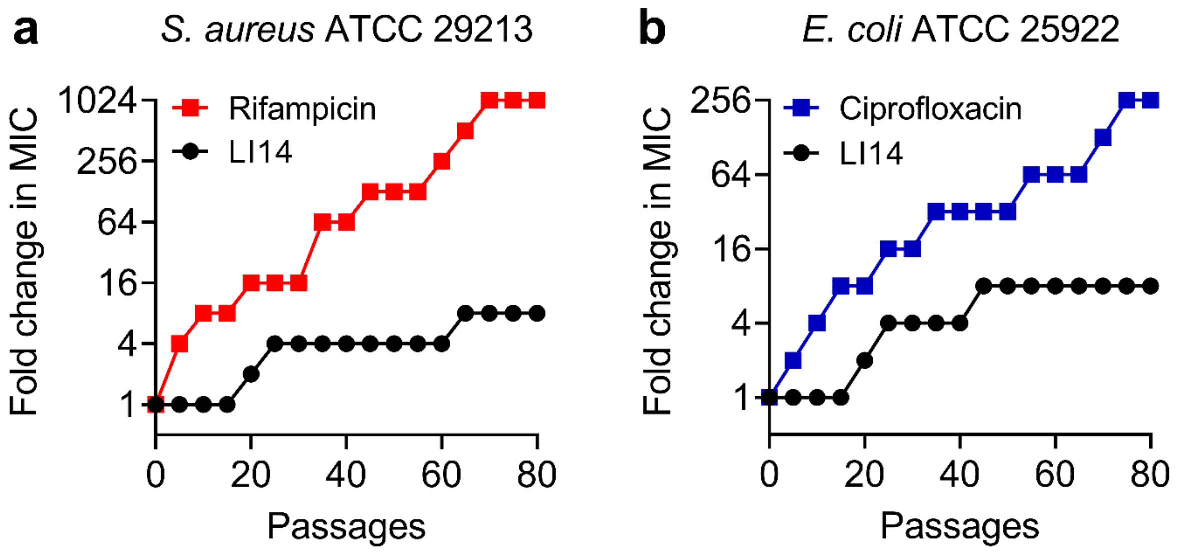

Supplementary Fig. 3 Bacterial pathogens display low propensity for resistance development to LI14 peptide.

Development of resistance assays for *S. aureus* ATCC 29213 **(a)** and *E. coli* ATCC 25922 **(b)** during 80 serial passages in the presence of sub-inhibitory concentrations of LI14, rifampicin or ciprofloxacin in MHB. Rifampicin and ciprofloxacin were used as positive controls. Data are representative of three biological replicates.

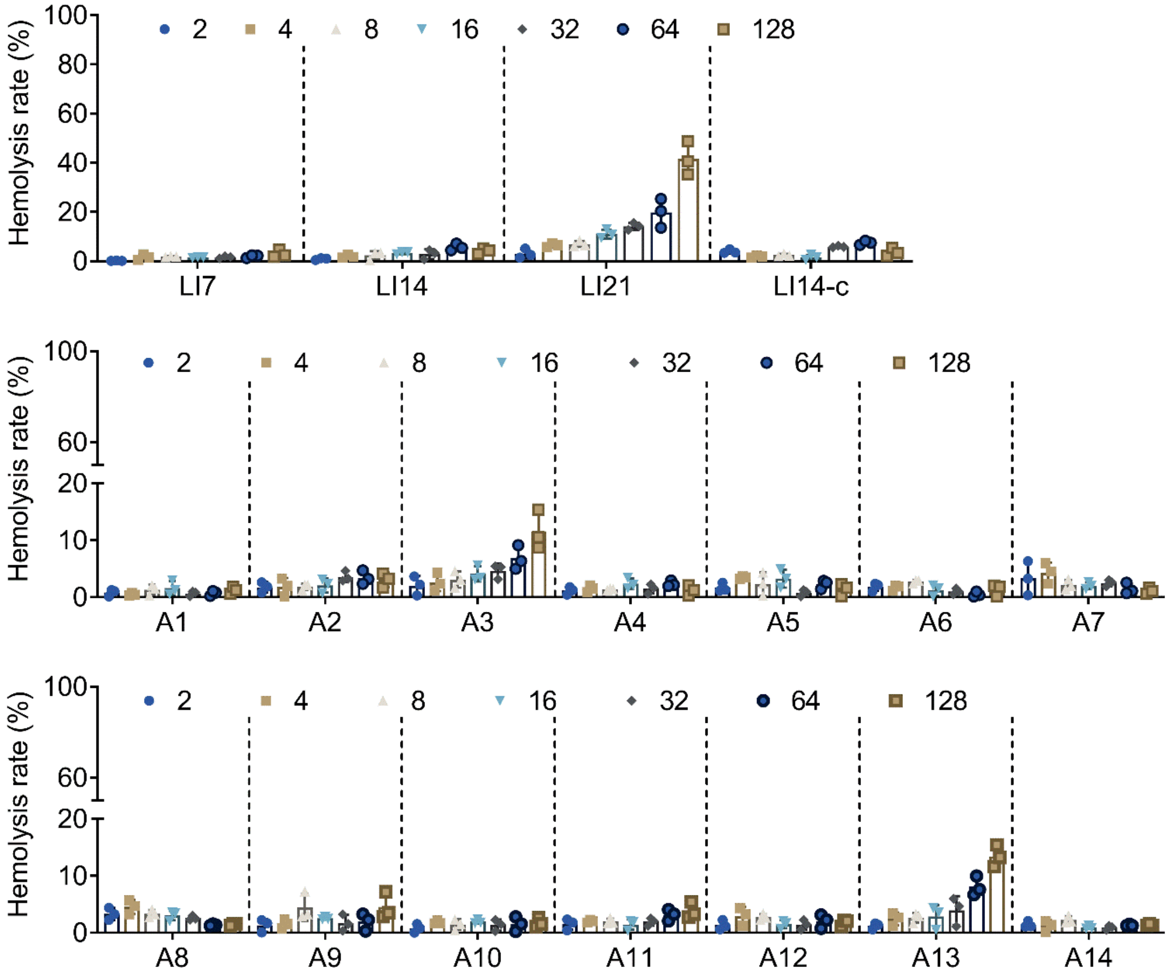

Supplementary Fig. 4 Hemolysis analysis of AMPs and alanine substitutions of LI14 peptide.

Hemolysis activity toward mammalian red blood cells (RBCs). Water and PBS were used as positive (100% hemolysis) or negative (0% hemolysis) controls. Data were showed as mean ± SD from three biological replicates.

a

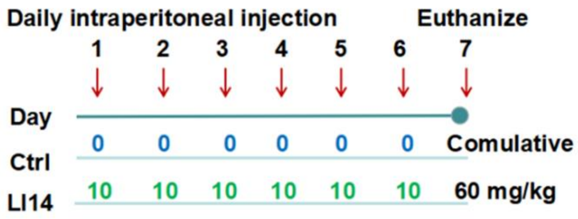

b

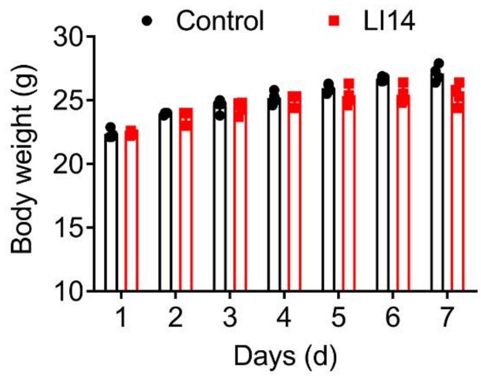

d

Parameters	Unit	Control	LI14
Glucose	mmol/L	4.11-7.94	9.7
Urea Nitrogen	mmol/L	2.5-9.6	5.97
Ca^{2+}	mmol/L	1.98-3	2.49
Creatinine	μmol/L	44-159	9.2
Total protein	g/L	52-82	57.1
Albumin (ALB)	g/L	23-40	29.0
Globulin (GLO)	g/L	25-45	28.1
ALB/GLO		1.2-2.3	1.0
Phosphorous	mmol/L	0.81-2.2	3.45
ALT	U/L	10-100	27.4
AST	U/L	0-50	27.4
AST/ALT		4.0	3.95

c

Parameters	Unit	Control	LI14
WBC	10^9 cells/L	6.1 (0.8-6.8)	6.0
Lymph#	10^9 cells/L	4.9 (0.7-5.7)	4.4
Mon#	10^9 cells/L	0.1 (0.0-0.3)	0.2
Gran#	10^9 cells/L	1.1 (0.1-1.8)	1.4
Lymph%	%	80.1 (55.8-90.6)	73.4
Mon%	%	2.3 (1.8-6.0)	3.1
Gran%	%	17.6 (8.6-38.96)	23.5
RBC	10^{12} cells/L	8.91 (6.39-9.42)	8.97
HGB	g/L	143 (110-143)	142
HCT	%	43.4 (34.6-44.6)	36.3
MCV	fL	48.8 (48.2-58.3)	40.5
MCH	pg	16.0 (15.8-19.0)	17.8
MCHC	g/L	329 (302-353)	440
RDW	%	14.7 (13.0-17.0)	13.8
PLT	10^9 cells/L	443 (450-1590)	164
MPV	fL	6.0 (3.8-6.0)	5.6
PDW		15.0	14.7
PCT	%	0.265	0.091

e

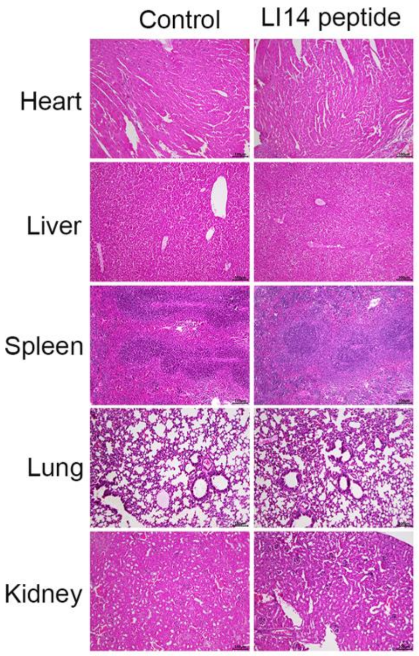

Supplementary Fig. 5 *In vivo* toxicity assessment of LI14 peptide.

(a) Scheme of the LI14 peptide toxicity study in mice. CD-1 female mice (n = 4 biologically independent animals per group) were intraperitoneally administered with LI14 (10 mg/kg) daily for a week. **(b-e)** Body weight **(b)**, whole-blood cell profiles **(c)**, serum biochemical

index **(d)** and histology images (H&E stained, **e**) of mice after treatment with LI14 for a week. Scale bar, 100 μm.

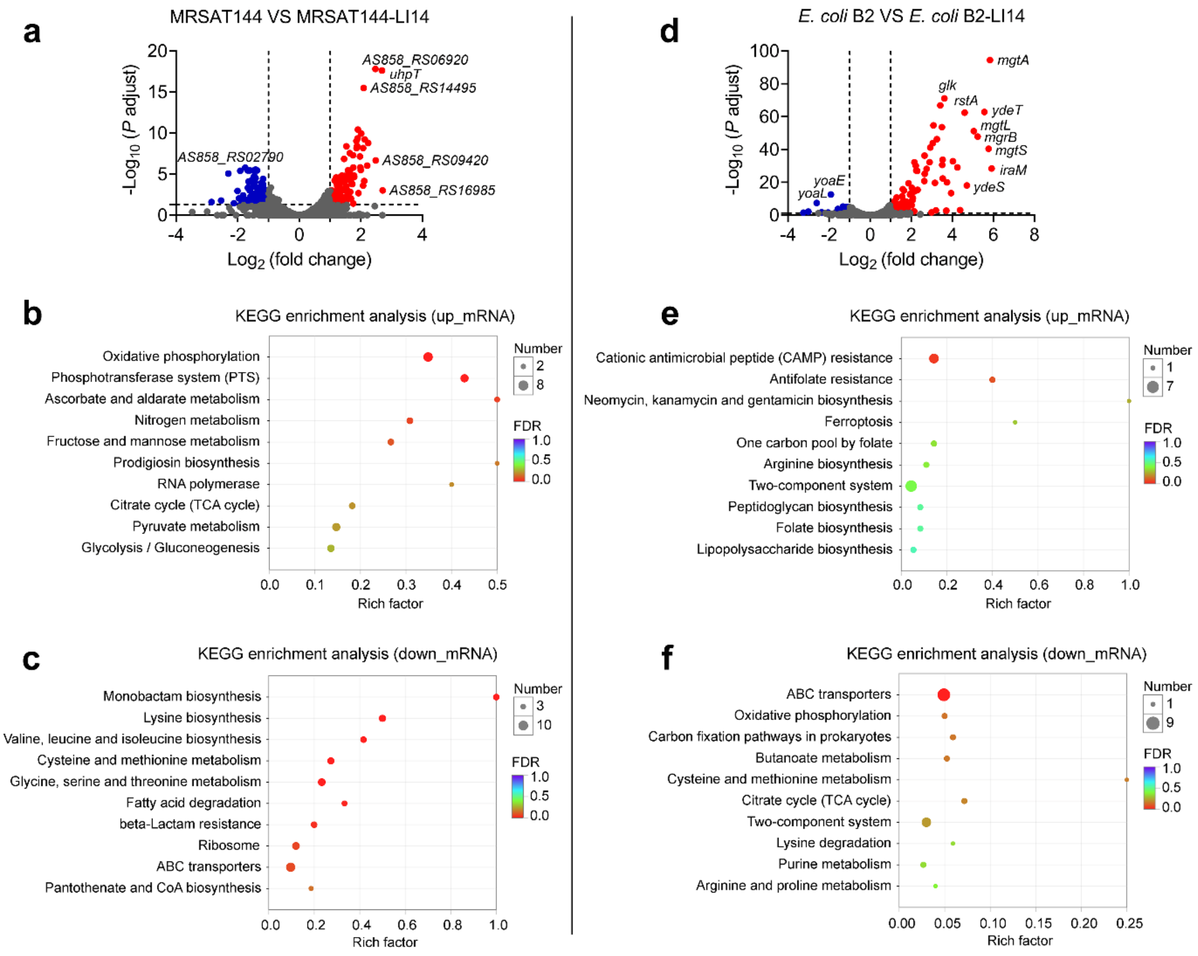

Supplementary Fig. 6 Transcriptional profile of MRSA T144 or *E. coli* B2 exposed to LI14 peptide.

(a, d) Volcano plot of the differential expression genes (DEGs) in MRSA T144 **(a)** or *E. coli* B2 **(d)** treated by LI14 peptide (8-fold MIC) for 4 h. The x- and y-axes represent the expression changes and corresponding statistically significant degree, respectively.

(b, c) KEGG (Kyoto Encyclopedia of Genes and Genomes) enrichment analysis of up-regulated DEGs **(b)** and down-regulated DEGs **(c)** in MRSA T144. The most significant enriched pathways are shown.

(e, f) KEGG enrichment analysis of up-regulated DEGs **(e)** and down-regulated DEGs **(f)** in *E. coli* B2. The most significant enriched pathways are shown.

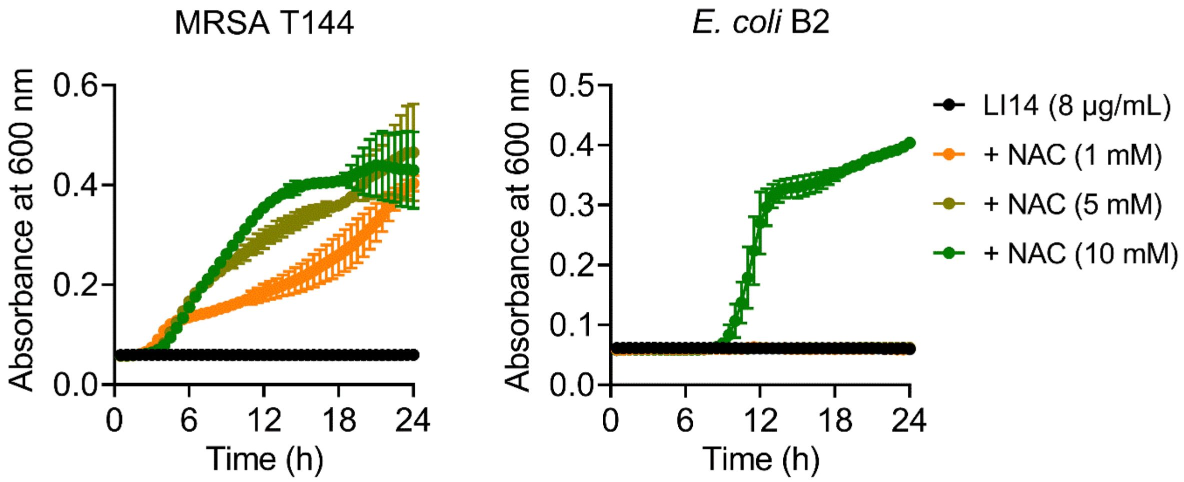

Supplementary Fig. 7 *N*-acetyl-L-cysteine (NAC) abolishes the antibacterial activity of LI14 peptide.

Growth curves of MRSA T144 and *E. coli* B2 in the presence of LI14 (8 μg/mL) alone or in combination with varying concentrations of NAC during 24 h were determined by monitoring the absorbance of cultures at 600 nm. Data were showed as mean ± SD from three biological replicates.

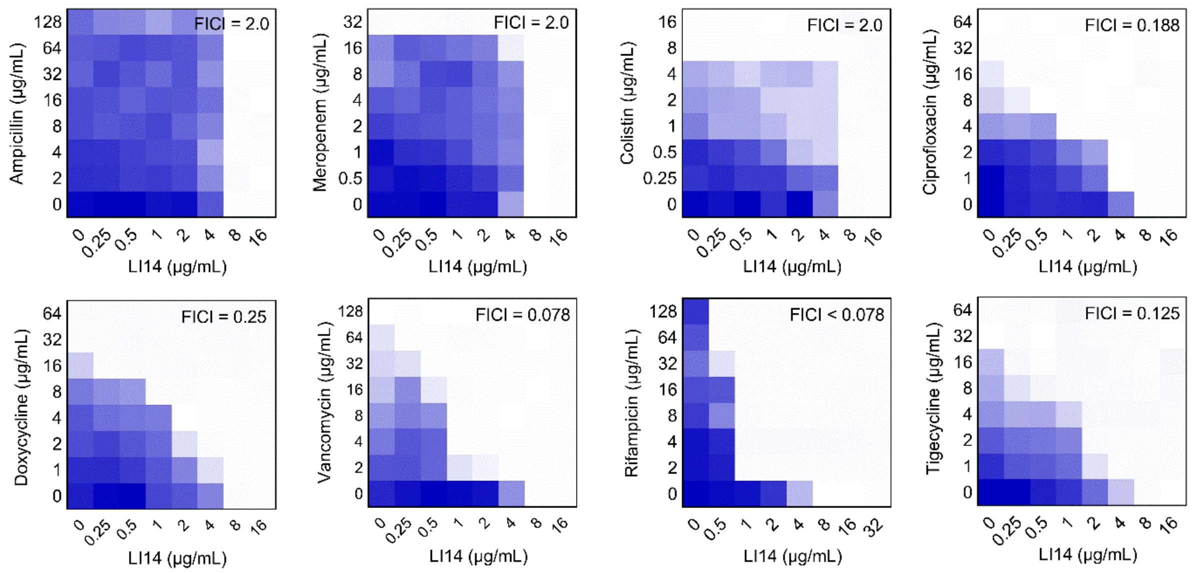

Supplementary Fig. 8 LI14 effectively potentiates multi-classes of antibiotic against MDR *E. coli* B2 or *E. coli* B3-1 (only for tigecycline).

Checkboard assay of LI14 in combination with ampicillin, meropenem, colistin, ciprofloxacin, doxycycline, vancomycin, rifampicin and tigecycline against *E. coli* B2 or *E. coli* B3-1. Dark blue represents greater growth, related to Table S1. Data represent the mean of three biological replicates.

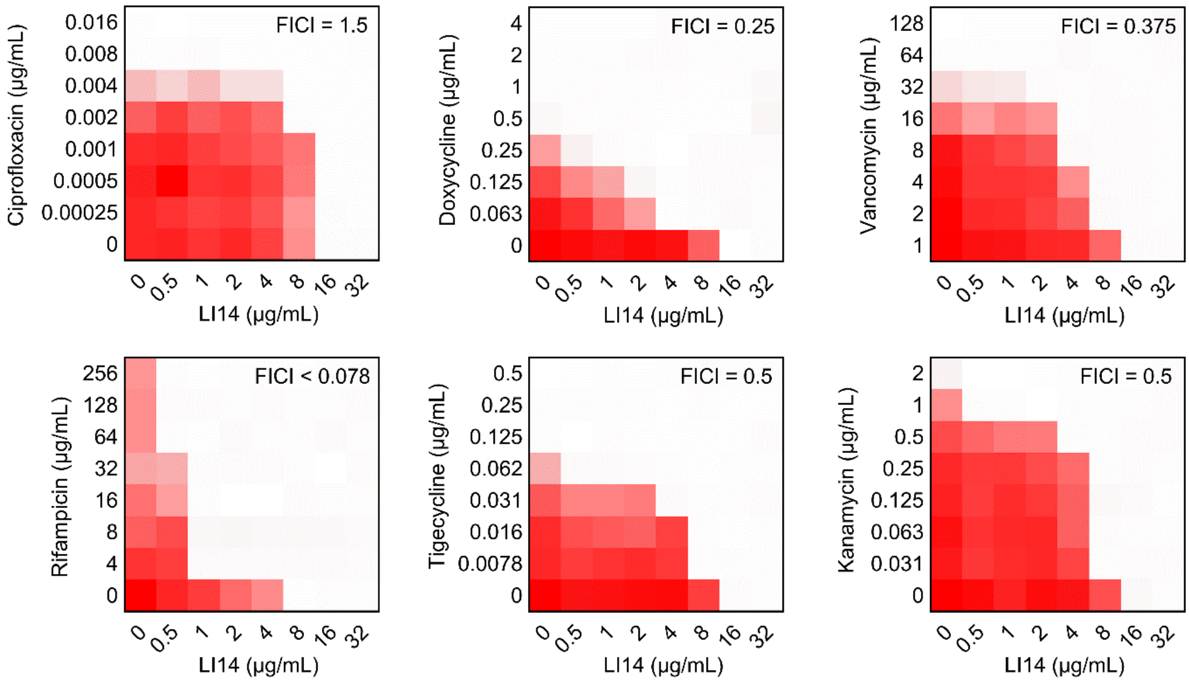

Supplementary Fig. 9 LI14 potentiates multi-classes of antibiotic against *E. coli* ATCC 25922.

Checkboard assay of LI14 in combination with ciprofloxacin, doxycycline, vancomycin, rifampicin, tigecycline and kanamycin against *E. coli* ATCC 25922. Dark red represents greater growth. Data represent the mean of three biological replicates.

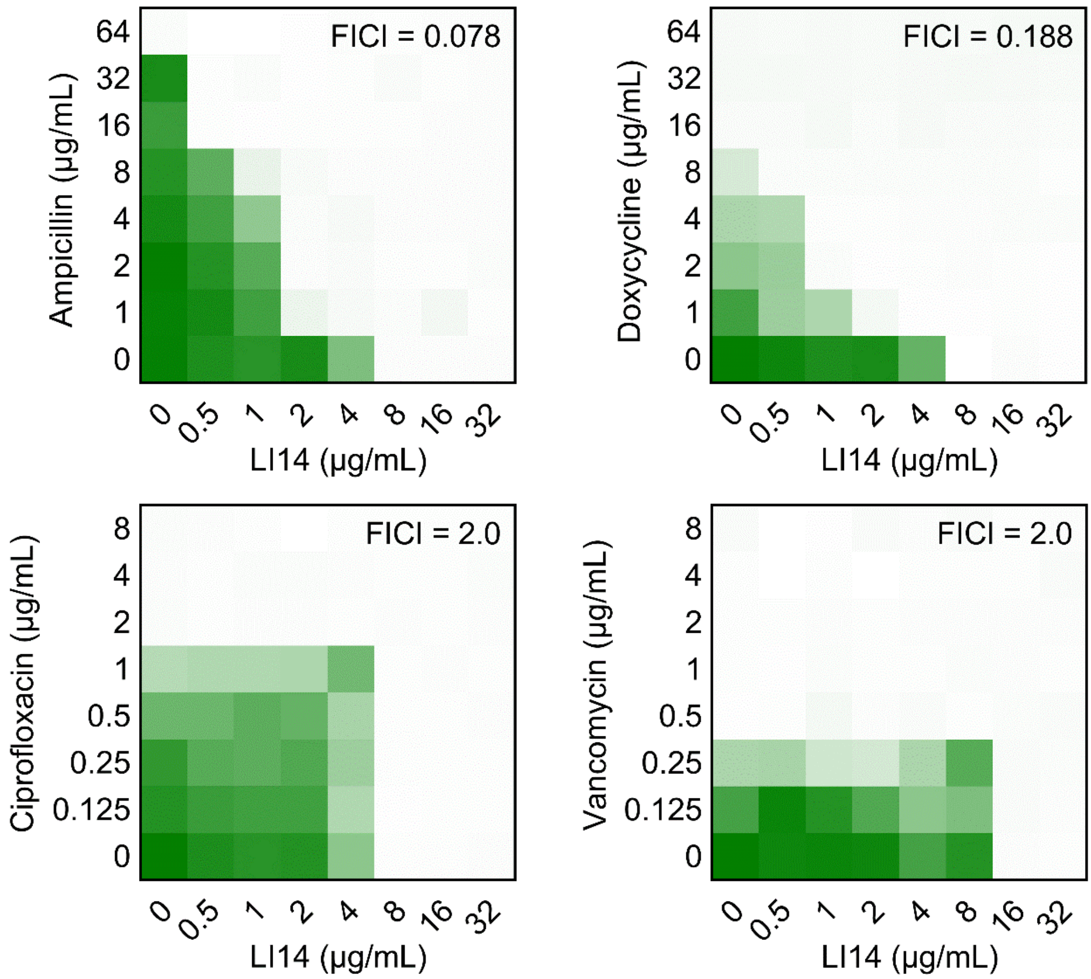

Supplementary Fig. 10 LI14 potentiates ampicillin and doxycycline against MRSA T144. Checkboard assay of LI14 in combination with ampicillin, doxycycline, ciprofloxacin and vancomycin against MRSA T144. Dark green represents greater bacterial density. Data represent the mean of three biological replicates.

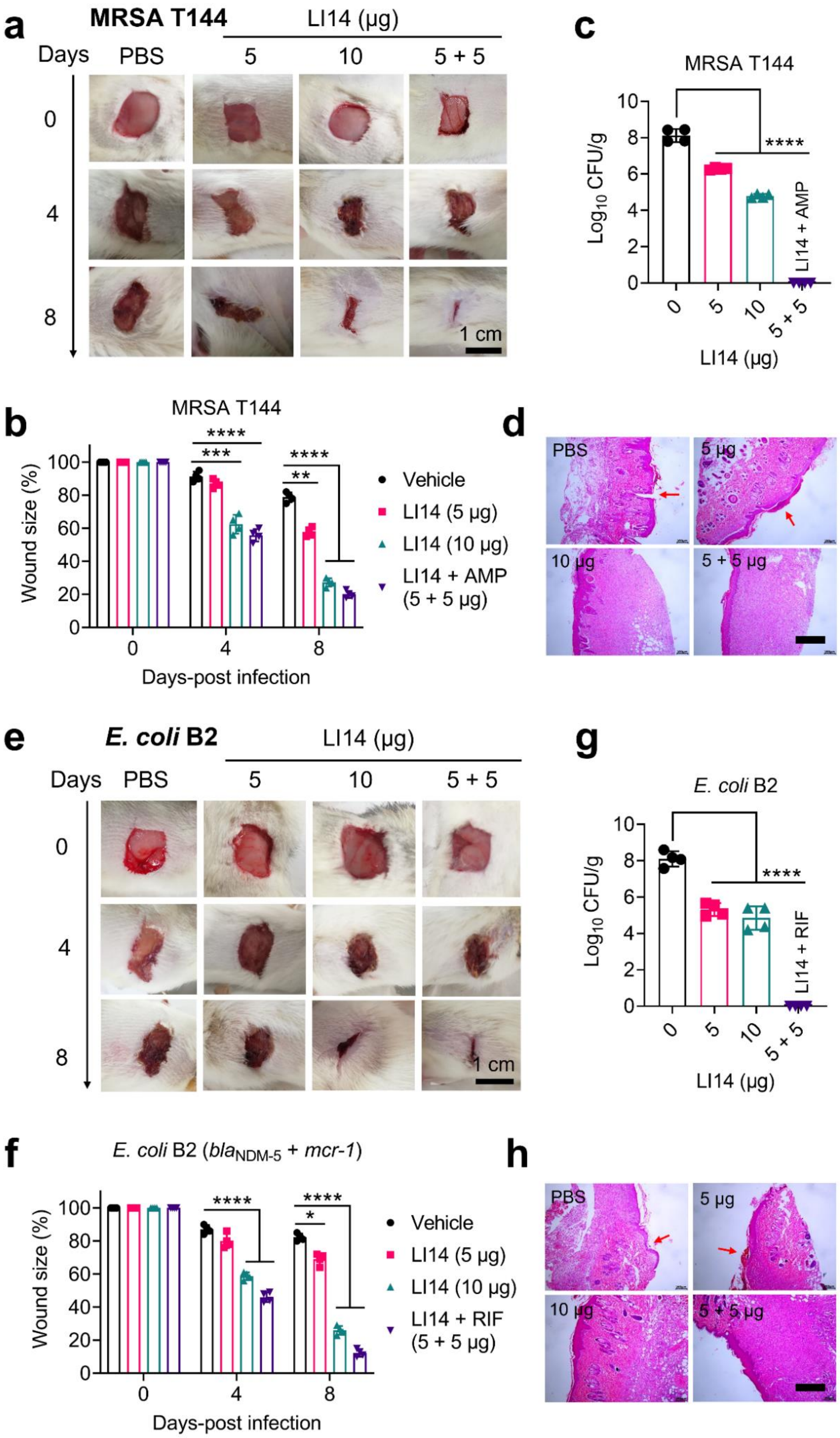
a
MRSA T144
LI14 (μg)
Days PBS 5 10 5 + 5
0
4
8
1 cm
c
MRSA T144
Log10 CFU/g
LI14 + AMP
****
0 5 10 5 + 5
LI14 (μg)
b
MRSA T144
Wound size (%)
****
***
****
**
Days-post infection
Vehicle
LI14 (5 μg)
LI14 (10 μg)
LI14 + AMP (5 + 5 μg)
d
PBS
5 μg
10 μg
5 + 5 μg
e
E. coli B2
LI14 (μg)
Days PBS 5 10 5 + 5
0
4
8
1 cm
g
E. coli B2
Log10 CFU/g
****
LI14 + RIF
0 5 10 5 + 5
LI14 (μg)
f
E. coli B2 (blaNDM-5 + mcr-1)
Wound size (%)
****
****
*
Days-post infection
Vehicle
LI14 (5 μg)
LI14 (10 μg)
LI14 + RIF (5 + 5 μg)
h
PBS
5 μg
10 μg
5 + 5 μg

Supplementary Fig. 11 LI14 exhibits potent *in vivo* efficacy in rat wound infection models. **(a-d)** Mouse skin wound infection models by MRSA T144 (n = 4 biologically independent animals per group). **a** Photographs of wounds treated with LI14 (5 μg and 10 μg) or in combination with ampicillin (5 + 5 μg) for 0, 4 and 8 days (scale bar, 1 cm). **(b)** The wound size at 0th, 4th and 8th day after infection by MRSA T144. **(c)** The subeschar bacterial colonies at 8th day. **(d)** H.E. staining for the histological change of skin wound at 8th day (scale bar, 500 μm).

(e-h) Mouse skin wound infection models by *E. coli* B2 (n = 4 biologically independent animals per group). **(e)** Photographs of wounds treated with LI14 (5 μg and 10 μg) or in combination with rifampicin for (5 + 5 μg) for 0, 4 and 8 days (scale bar, 1 cm). **(f)** The wound size at 0th, 4th and 8th day after infection by *E. coli* B2. **(g)** The subeschar bacterial colonies at 8th day. **(h)** H.E. staining for the histological change of skin wound at 8th day (scale bar, 500 μm).

Data in **b, c, f and g** were showed as mean ± SD and analyzed by one-way ANONA (*$P < 0.05$, **$P < 0.01$, ***$P < 0.001$, ****$P < 0.0001$).

.

Tables

Supplementary Table 1 Bacteria strains used in this study.

Strains	Source/Reference
**Gram-positive bacteria**	
S. aureus ATCC 29213	ATCC
MRSA T144	In this study
MRSA 1518	In this study
MRSA 1530	In this study
S. aureus 215 (*cfr* + LZD^R)	In this study
S. aureus G16 (RIF^R)	In this study
E. faecalis A4 (VRE, VanA)	In this study
E. faecalis 1F-1	In this study
E. faecium 5F-10	In this study
**Gram-negative bacteria**	
E. coli ATCC 25922	ATCC
E. coli B2 ($\textit{bla}_{\text{NDM-5}}$ + *mcr-1*)	In this study
E. coli C3 ($\textit{bla}_{\text{NDM-1}}$)	In this study
E. coli G6 ($\textit{bla}_{\text{NDM-5}}$)	In this study
E. coli G92 (*mcr-1*)	In this study
E. coli CP131 (*mcr-3*)	In this study
E. coli B3-1 (*tet*(X4))	In this study
E. coli 1F28 (*tet*(X4))	In this study
S. enteritidis ATCC 13076	ATCC
A. baumannii ATCC 19609	ATCC
A. baumannii C222 (*tet*(X6))	In this study
P. aeruginosa PA14	In this study
K. pneumoniae ATCC 700603	ATCC
K. pneumoniae D120 (*mcr-8*)	In this study
P. cibarius HNCF44W ($\textit{bla}_{\text{NDM-1}}$+ *tet*(X6))	In this study
E. coli MG1655	In this study
E. coli MG1655 (Δ*gltA*)	In this study
E. coli MG1655 (Δ*cydB*)	In this study
E. coli MG1655 (Δ*katE*)	In this study

ATCC, American Type Culture Collection; RIF, rifampicin; VRE, vancomycin-resistant enterococci.

Supplementary Table 2 Physicochemical parameters of LI AMPs.

Name	Sequence ($N \rightarrow C$)	Formula	MW	Net charge	H[a]	μH[b]	pI[c]	Purity (%)
LI7	LKKLCRI-NH2	$C_{39}H_{76}N_{12}O_8S_1$	873.17	+3	0.570	--	10.06	96.69%
LI14	LKKLCRILKKLCRI-NH2	$C_{78}H_{150}N_{24}O_{15}S_2$	1728.32	+6	0.570	0.811	10.48	95.47%
LI21	LKKLCRILKKLCRILKKLCRI-NH2	$C_{117}H_{224}N_{36}O_{22}S_3$	2583.47	+9	0.570	0.790	10.66	96.31%
LI14 (S-S)	LKKLc[CRILKKLC]RI-NH2	$C_{78}H_{149}N_{25}O_{14}S_2$	1725.34	+6	0.570	0.811	10.48	95.86%
LI14-A1	AKKLCRILKKLCRI-NH2	$C_{75}H_{145}N_{25}O_{14}S_2$	1685.26	+6	0.570	0.744	10.48	96.36%
LI14-A2	LAKLCRILKKLCRI-NH2	$C_{75}H_{144}N_{24}O_{14}S_2$	1670.25	+5	0.640	0.736	10.33	99.65%
LI14-A3	LKALCRILKKLCRI-NH2	$C_{75}H_{146}N_{24}O_{14}S_2$	1670.25	+5	0.640	0.776	10.33	95.67%
LI14-A4	LKKACRILKKLCRI-NH2	$C_{75}H_{145}N_{25}O_{14}S_2$	1685.26	+6	0.570	0.715	10.48	96.46%
LI14-A5	LKKLARILKKLCRI-NH2	$C_{78}H_{151}N_{25}O_{14}S_1$	1695.29	+6	0.570	0.808	11.17	95.98%
LI14-A6	LKKLCAILKKLCRI-NH2	$C_{75}H_{144}N_{22}O_{14}S_2$	1642.23	+5	0.640	0.717	10.07	96.01%
LI14-A7	LKKLCRALKKLCRI-NH2	$C_{75}H_{145}N_{25}O_{14}S_2$	1685.26	+6	0.570	0.789	10.48	95.76%
LI14-A8	LKKLCRIAKKLCRI-NH2	$C_{75}H_{145}N_{25}O_{14}S_2$	1685.26	+6	0.570	0.722	10.48	97.84%
LI14-A9	LKKLCRILAKLCRI-NH2	$C_{75}H_{144}N_{24}O_{14}S_2$	1670.25	+5	0.640	0.761	10.33	96.68%
LI14-A10	LKKLCRILKALCRI-NH2	$C_{75}H_{144}N_{24}O_{14}S_2$	1670.25	+5	0.640	0.748	10.33	95.14%
LI14-A11	LKKLCRILKKACRI-NH2	$C_{75}H_{145}N_{25}O_{14}S_2$	1685.26	+6	0.570	0.731	10.48	96.7%
LI14-A12	LKKLCRILKKLARI-NH2	$C_{78}H_{151}N_{25}O_{14}S_1$	1695.29	+6	0.570	0.778	11.17	96.66%
LI14-A13	LKKLCRILKKLCAI-NH2	$C_{75}H_{144}N_{22}O_{14}S_2$	1642.23	+5	0.640	0.720	10.07	96.15%
LI14-A14	LKKLCRILKKLCRA-NH2	$C_{75}H_{145}N_{25}O_{14}S_2$	1685.26	+6	0.570	0.826	10.48	95.96%

[a]Total hydrophobic ratio (%), calculated from http://aps.unmc.edu/AP/prediction/prediction_main.php.

[b]Relative hydrophobic moment (μHrel) values were employed to analyze the level of amphipathicity of all peptides and were calculated from http://heliquest.ipmc.cnrs.fr/cgi-bin/ComputParams.py.

[c]The isoelectric point (pI) values of derivatives were determined by ExPASy (http://web.expasy.org/compute_pi/).

Supplementary Table 3 Antibacterial activity of LI14 derivates against MRSA T144 and *E. coli* B2.

AMPs	*S. aureus* 29213	MRSA T144	*E. coli* 25922	*E. coli* B2
LI14 (S-S)	4	4	8	4
LI14-A1	16	64	16	4
LI14-A2	8	16	8	4
LI14-A3	8-16	8	16	8
LI14-A4	64	64	16-32	8
LI14-A5	16	16	4	4
LI14-A6	>128	>128	128	32
LI14-A7	64	64	32	16
LI14-A8	128	64	16	8
LI14-A9	16	16	8-16	4
LI14-A10	16-32	16	16-32	8
LI14-A11	64	64	16	8
LI14-A12	8	16	2	4
LI14-A13	16	16	8	4
LI14-A14	16	32	16	4

Supplementary Table 4 Thermal, pH, salts and protease stability of AMPs against MRSA T144 and *E. coli* B2 (MIC, μg/mL).

Treatment	MRSA T144			*E. coli* B2		
	L14	L21	L14 (S-S)	L14	L21	L14 (S-S)
Control	4	16	4	4	16	4
Temperature						
40℃	4	16	4	4	16	4
60℃	4	16	4	4	16	4
80℃	4	16	4	4	16	4
100℃	8	32	8	8	16	8
121℃	16	32	16	4	16	8
pH						
2	8	16	4	4	16	4
4	8	16	4	4	16	4
6	8	16	4	4	16	4
8	4	16	4	4	16	4
10	8	16	16	4	16	8
12	4	16	16	4	16	8
Salts (10 mM)						
NaCl	8	16	4	4	16	4
KCl	8	16	4	4	16	4
$MgCl_2$	32	32	16	128	64	32
Protease (1 mg/mL)						
Pepsin	4	16	4	8	16	8
Trypsin	>128	>128	>128	>128	>128	>128
Papain	>128	>128	>128	>128	>128	>128
Serum (10%)	32	64	16	4	32	4
DMEM (10%)	16	16	4	8	16	4

Supplementary Table 5 Synergistic activity of LI14 in combination with different classes of antibiotic against MDR *E. coli* B2 or tigecycline-resistant *E. coli* B3-1.

Targets	Antibiotics	MIC[a] (μg/mL)	FIC index	MIC[b] (μg/mL)	Potentiation (fold)[c]
Cell wall	Ampicillin	>128	2	>128	–
	Meropenem	32	2	32	–
	Vancomycin	128	0.078	2	64
Cell membrane	Colistin	8	2	8	–
DNA synthesis	Ciprofloxacin	32	0.188	4	8
RNA synthesis	Rifampicin	>128	<0.078	2	>64
Protein	Doxycycline	32	0.25	4	8
	Tigecycline	32	0.125	2	16

[a/b]MICs of antibiotics in the absence or presence of ¼ MIC of LI14.

[c]Degree of antibiotics potentiation in the presence of ¼ MIC of LI14.

–, none of potentiation activity.

Supplementary Table 6 Synergistic activity of LI14 in combination with different classes of antibiotics against sensitive *E. coli* ATCC 25922.

Antibiotics	MIC[a] (μg/mL)	FIC index	MIC[b] (μg/mL)	Potentiation (fold)[c]
Ciprofloxacin	0.008	1.5	0.008	–
Doxycycline	0.5	0.25	0.063	8
Vancomycin	64	0.375	8	8
Rifampicin	>128	<0.078	2	>64
Tigecycline	0.063	0.5	0.016	4
Kanamycin	2	0.5	0.5	4

[a/b]MICs of antibiotics in the absence or presence of ¼ MIC of LI14.

[c]Degree of antibiotics potentiation in the presence of ¼ MIC of LI14.

–, none of potentiation activity.

Supplementary Table 7 Synergistic activity of LI14 in combination with Gram-positive antibiotics against MRSA T144.

Antibiotics	MIC[a] (μg/mL)	FIC index	MIC[b] (μg/mL)	Potentiation (fold)[c]
Ampicillin	64	0.078	1	64
Doxycycline	16	0.188	1	16
Ciprofloxacin	2	2.0	2	–
Vancomycin	0.5	2.0	0.5	–

[a/b]MICs of antibiotics in the absence or presence of ¼ MIC of LI14.

[c]Degree of antibiotics potentiation in the presence of ¼ MIC of LI14.

–, none of potentiation activity.
